# Synergistic inhibition of *Streptococcus mutans* biofilms by fluoride and epigallocatechin gallate: insights from multi-omics analysis

**DOI:** 10.3389/fmicb.2026.1766833

**Published:** 2026-02-19

**Authors:** Yuanyuan Chen, Tiantian Wu, Long Jiang, Junjun Zhao

**Affiliations:** 1 Department of General Dentistry, School of Medicine, Shanghai Ninth People’s Hospital, Shanghai Jiao Tong University, Shanghai, China; 2 National Center for Stomatology, School of Medicine, Shanghai Ninth People’s Hospital, Shanghai Jiao Tong University, Shanghai, China; 3 National Clinical Research Center for Oral Diseases, College of Stomatology, School of Medicine, Shanghai Ninth People’s Hospital, Shanghai Jiao Tong University, Shanghai, China; 4 Shanghai Key Laboratory of Stomatology, Shanghai Research Institute of Stomatology, School of Medicine, Shanghai Ninth People’s Hospital, Shanghai Jiao Tong University, Shanghai, China; 5 Shanghai Key Laboratory of Translational Medicine on Ear and Nose Diseases, Shanghai, China

**Keywords:** biofilms, epigallocatechin gallate, fluoride, omics, *Streptococcus mutans*

## Abstract

**Introduction:**

*Streptococcus mutans* biofilms are central to the development of dental caries, and strategies that effectively attenuate biofilm formation remain essential for disease control. This study examined the combined antibiofilm effects of sodium fluoride (NaF) and epigallocatechin gallate (EGCG), a green tea–derived polyphenol, and delineated the underlying mechanisms using an integrative multi-omics framework.

**Materials and methods:**

Synergistic interactions between NaF and EGCG were first assessed by crystal violet staining and checkerboard microdilution analysis. Biofilm biomass, viability, and matrix composition were quantified using colony-forming unit assays, scanning electron microscopy, confocal laser scanning microscopy, and measurements of water-insoluble extracellular polysaccharides. Transcriptomic, proteomic, and metabolomic profiling were carried out to identify perturbed pathways, and arginine levels were quantified to evaluate metabolic responses.

**Results:**

Co-treatment with NaF and EGCG produced a synergistic inhibitory effect, markedly reducing biomass, viable cells, and extracellular polysaccharide content. Microscopic analyses demonstrated disrupted microcolony organization and compromised matrix architecture. Multi-omics profiling revealed concentration-dependent alterations in amino acid, carbohydrate metabolism, nucleotide and energy-related processes.

**Conclusion:**

NaF and EGCG synergistically disrupt *S. mutans* biofilms by simultaneously impairing extracellular polysaccharide synthesis, redox balance, and central metabolic activity. These findings support the potential of combining EGCG with fluoride to enhance caries prevention strategies.

## 1 Introduction

Dental caries is the most widespread biofilm-mediated infectious condition in humans and continues to impose substantial clinical and economic burdens worldwide (Frencken et al., 2017; Kazeminia et al., 2020; Pitts et al., 2017). Epidemiological data indicate that a substantial proportion of both children and adults are affected by dental caries, which negatively impacts not only physical health, such as compromised dental aesthetics particularly in anterior teeth, but also mental well-being due to associated pain and discomfort. Consequently, identifying affordable and effective strategies for caries prevention and management remains a critical public health priority.

Within the complex environment of dental cavities, *Streptococcus mutans* (*S. mutans*) plays a pivotal role as a primary cariogenic pathogen (Lemos et al., 2019). It contributes to biofilm virulence through the synthesis of insoluble glucans, forming a dense extracellular polysaccharide (EPS) matrix that facilitates bacterial adhesion, accumulation, and maintenance of the structural integrity of the biofilm (Lemos et al., 2019). Therefore, agents capable of disrupting EPS production and biofilm formation represent promising antimicrobial candidates for caries control.

Fluoride remains the cornerstone of anticaries strategies. Its primary effects include promoting enamel remineralization, inhibiting demineralization, and facilitating the formation of fluorapatite, which displays greater acid resistance than hydroxyapatite (Featherstone, 1999). In addition, fluoride enhances calcium and phosphate deposition under cariogenic conditions and exerts antimicrobial activity by blocking enolase-mediated glycolysis, reducing glucose uptake (Buzalaf et al., 2011; Van Loveren, 2001), as well as impairing proton translocation essential for microbial energy production (Marquis, 1995). These actions collectively attenuate biofilm formation (Melkam et al., 2024; Liu et al., 2012).

However, continuous fluoride exposure may select for fluoride-tolerant *S. mutans* strains, accompanied by metabolic alterations and potential disturbances in oral microbial ecology (Shen et al., 2022). These concerns have led to intensified efforts toward strategies that augment fluoride efficacy using synergistic antimicrobial agents (Koo et al., 2006; Dehghani et al., 2015; Zheng et al., 2015). Combinations of fluoride with adjuncts such as arginine, chlorhexidine, or zinc enhance suppression of *S. mutans* by acting on distinct microbial targets, potentially reducing the emergence of resistance. Nonetheless, long-term use of broad-spectrum antimicrobials raises concerns about ecological disruption, emphasizing the need for safer enhancers of fluoride activity (Koo et al., 2006; Dehghani et al., 2015; Zheng et al., 2015), emphasizing the need for safer enhancers of fluoride activity.

Plant-derived antimicrobials have gained considerable attention as biocompatible alternatives, particularly in oral care applications. Green tea polyphenols are routinely incorporated into toothpastes and mouthrinses (Aragao et al., 2022). Epigallocatechin gallate (EGCG), the most abundant and bioactive catechin in green tea, has been reported to inhibit *S. mutans* biofilm formation. Nevertheless, optimal dosing is controversial, with effective concentrations of EGCG ranging from 15.6 μg/mL to 2 mg/mL across different studies (Aragao et al., 2022; Han et al., 2021; Xu et al., 2011; Schneider-Rayman et al., 2021; Hairul Islam et al., 2020). These discrepancies hinder the establishment of standardized formulations for clinical translation.

Evidence regarding the combined use of fluoride and EGCG remains limited. Only one previous study examined this combination, focusing on acid production rather than biofilm formation and employing exposure durations too short to represent mature biofilm development (Han et al., 2023). Moreover, the fluoride species used did not reflect those commonly applied in clinical settings (Han et al., 2023). Acidogenesis represents only one downstream outcome of a fully developed biofilm, whereas the EPS matrix dictates ecological stability, diffusion barriers, and antimicrobial tolerance. Therefore, assessing biofilm inhibition provides a more comprehensive indicator of anticariogenic potential. The interactions between fluoride and EGCG during biofilm development, as well as clinically relevant concentration thresholds, therefore require systematic clarification.

In this context, the present study aimed to define optimal concentrations and elucidate the molecular basis of NaF and EGCG co-treatment using a multi-omics approach. By integrating phenotypic assays with transcriptomic, proteomic, and metabolomic analyses, this work provides mechanistic insight into how these agents jointly impair *S. mutans* biofilms and supports their potential application in caries prevention.

## 2 Materials and methods

### 2.1 Bacterial strain, culture conditions, and chemicals

*S. mutans* UA159 (ATCC 700610) was routinely cultivated in brain heart infusion (BHI; Difco, USA) at 37 °C under anaerobic conditions (85% N₂, 10% H₂, 5% CO₂). Epigallocatechin gallate (EGCG; MedChemExpress, China) was dissolved in 10% DMSO to generate stock solutions, followed by dilution in sterile water. The final DMSO concentration was maintained at 1% in all experimental and control groups. Sodium fluoride (NaF; Beijing Pufei Biotechnology Co., Ltd., China) was used as the fluoride source, with concentrations reported based on fluoride content. For initial inhibitory screening, EGCG and fluoride were evaluated at final concentrations ranging from 0.03–2 mg/mL and 3.9–500 ppm, respectively.

### 2.2 Biofilm inhibition assay

The inhibitory effects of fluoride or EGCG alone were assessed using a modified crystal violet staining assay to determine effective concentration ranges for subsequent synergy testing (Zhang et al., 2019; Hirasawa et al., 2006). Overnight cultures of *S. mutans* were diluted 1:1000 in BHI supplemented with 2% sucrose to yield approximately 2 × 10^6^ CFU/mL. Equal volumes of fluoride and EGCG were added at the indicated concentrations and incubated anaerobically for 24 h. Biofilms were washed three times with sterile water, fixed in absolute methanol, stained with 0.1% crystal violet for ≥15 min, rinsed, and solubilized in 95% ethanol. Biomass was quantified at OD₅₉₅ (BioTek, USA). All measurements were performed in triplicate. Relative inhibition was calculated as previously described (Dzoyem et al., 2025):


$$\text{inhibition}\;(\%)=(1-\frac{{\mathrm{OD}}_{\text{experiment}}-{\mathrm{OD}}_{\text{blank}}}{{\mathrm{OD}}_{\text{control}}-{\mathrm{OD}}_{\text{blank}}})*100\%$$

### 2.3 Checkerboard microdilution assay

**Figure 1:**
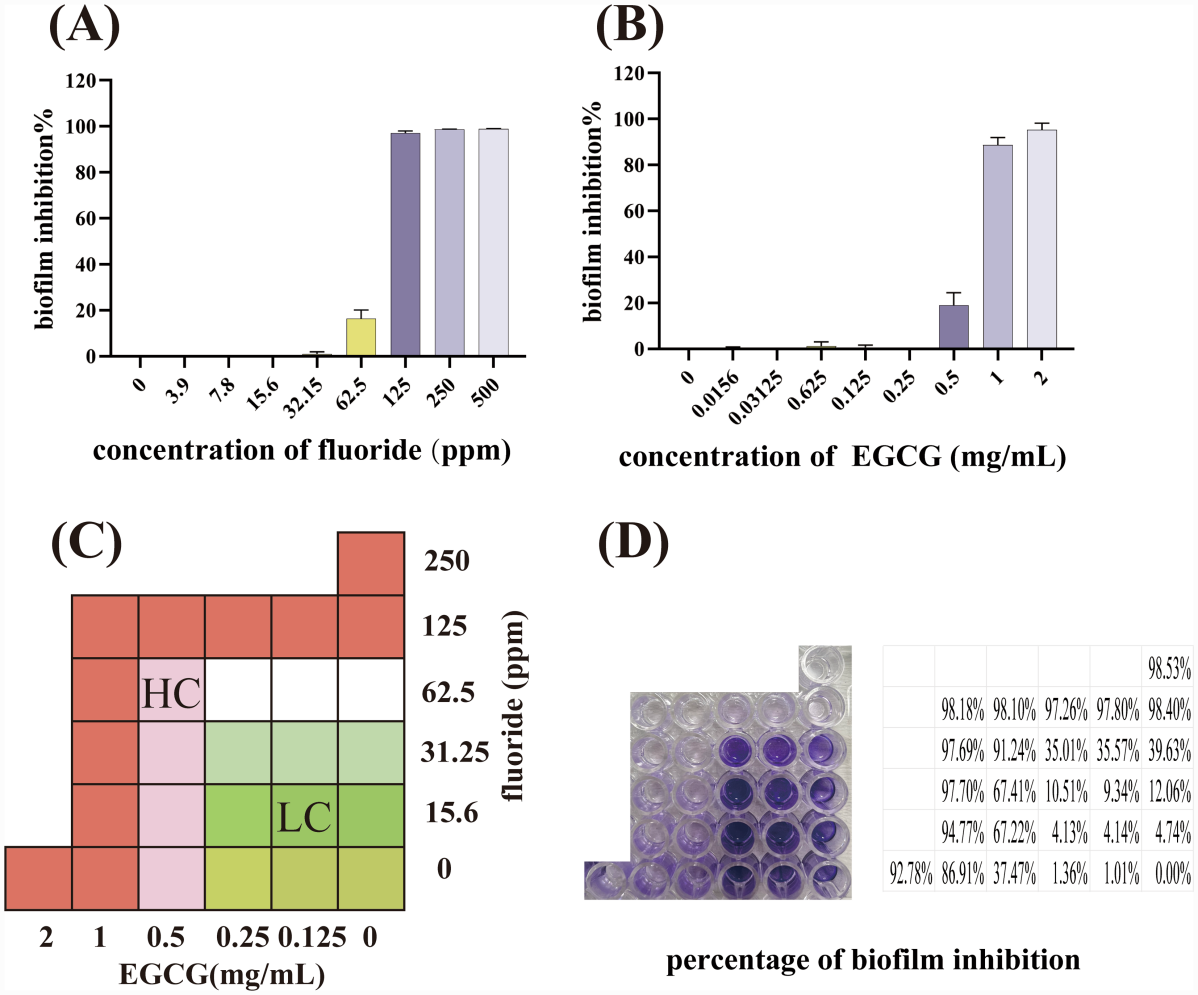
Effects of NaF and EGCG on *S. mutans* biofilm. **(A)** Percentage of *S. mutans* biofilms inhibition when treated with NaF. **(B)** Percentage of *S. mutans* biofilms inhibition when treated with EGCG. **(C)** Illustration of checkerboard microdilution assays on *S. mutans* biofilms. HC, high concentrations, 62.5 ppm of fluoride, 0.5 mg/mL of EGCG. LC, low concentrations, 15.6 ppm of fluoride, 0.125 mg/mL of EGCG. **(D)** Percentage of *S. mutans* biofilms inhibition combining with NaF and EGCG.

The combined effects of fluoride with EGCG were assessed by the checkerboard microdilution assay reported previously with some modifications (Zheng et al., 2015), and experiments were carried out in triplicate. Biofilm formation was nearly abolished when EGCG was applied at 2 mg/mL or fluoride at 250 ppm, whereas lower concentrations resulted in progressive biomass recovery (Figures 1A,B). Therefore, EGCG concentrations of 0.125–2 mg/mL and fluoride concentrations of 15.6–250 ppm was selected for synergy testing. Briefly, two-fold serial dilutions of NaF and EGCG were prepared along the ordinate and abscissa of 96-well microtiter plates, respectively. This configuration generated a concentration matrix allowing assessment of combined treatments across a range of dose combinations. Importantly, the marginal rows and columns of the checkerboard matrix contained wells with NaF alone or EGCG alone, respectively, thereby serving as internal single-agent controls within the same experimental setup. Equal volumes (100 μL each) of EGCG, fluoride, and bacterial inoculum were combined in 96-well plates and incubated for 24 h. Biomass was quantified by crystal violet staining. Fractional inhibitory concentration index (FIC) values were interpreted as synergy (≤0.5), indifference (0.5–4.0), or antagonism (>4.0). Based on these analyses, two treatment conditions were selected:

High-concentration (HC) group: 62.5 ppm fluoride + 0.5 mg/mL EGCG (biofilm inhibition >90%, FIC = 0.5).

Low-concentration (LC) group: 15.6 ppm fluoride + 0.125 mg/mL EGCG (minimal inhibition).

### 2.4 Growth of planktonic bacteria

Bactericidal effects under HC or LC conditions were quantified by colony-forming unit (CFU) enumeration (Chen et al., 2021). After 24 h treatment, cultures were serially diluted (10⁻¹–10⁻⁶), plated onto BHI agar, and incubated anaerobically for 36 h at 37 °C. CFU counts were obtained from three independent biological replicates.

### 2.5 SEM and CLSM imaging of biofilms

Biofilm morphology was evaluated using scanning electron microscopy (SEM; Zeiss Sigma 300) following established procedures (Chen et al., 2021). Biofilms grown on sterile coverslips for 24 h were fixed with 2.5% glutaraldehyde for ≥3 h, dehydrated through a graded ethanol series (30–100%), freeze-dried, sputter-coated with gold, and imaged at 1000×, 5,000×, and 10,000 × magnifications in three random fields per sample.

Three-dimensional architecture was examined using confocal laser scanning microscopy (CLSM; Zeiss LSM880) with a 63 × oil-immersion objective (Chen et al., 2021). SYTO9 and PI were excited at 488 nm and 543 nm, respectively. Z-stacks were collected at 3 μm intervals under constant gain and offset settings. Three biological samples per group were analyzed, with triplicate fields acquired from each.

### 2.6 Quantitative determination of water-insoluble EPS

Water-insoluble EPS was measured using the anthrone method (Cao et al., 2020). Biofilms grown in 12-well plates were harvested into PBS, centrifuged at 6000 × g for 10 min, and washed three times to remove soluble EPS. Pellets were incubated in 0.4 M NaOH for 2 h at 37 °C to extract alkali-soluble polysaccharides. Anthrone–sulfuric acid reagent was added, heated at 95 °C for 5 min, and absorbance was recorded at OD₆₂₅. CLSM visualization of EPS was performed using Alexa Fluor 647–conjugated Concanavalin A (Wu et al., 2020), followed by SYTO9 staining and imaging on a Leica Stellaris 8 confocal microscope using a 63 × oil objective.

### 2.7 Sample preparation for bioinformatics analysis

Biofilms were grown for 24 h in 6-well plates under control, LC, or HC conditions. After washing with PBS, biofilms were scraped and collected under aseptic conditions. Samples were divided into aliquots for transcriptomic RNA extraction, proteomic protein isolation, and metabolite preparation.

### 2.8 Transcriptome analysis

Total RNA (*n* = 4 biological replicates per group) was extracted, converted to cDNA, and sequenced (details in Supplementary Material). Gene abundance was normalized as FPKM. Differential gene expression analysis was performed using DESeq2 (padj ≤ 0.05, |log₂FC| ≥ 0) or edgeR (padj ≤ 0.005, |log₂FC| ≥ 1). GO (Gene Ontology) and KEGG (Kyoto Encyclopedia of Genes and Genomes) enrichment were conducted using clusterProfiler.

### 2.9 TMT (tandem mass tag) labeling proteomic analysis

Protein extraction and TMT labeling were performed on four biological replicates per group (details in Supplementary Material). Mass spectrometry–based quantification was conducted using a database search against the *S. mutans* UA159 protein sequence dataset retrieved from the NCBI database. Differentially expressed proteins (DEPs) were identified based on thresholds of *p* < 0.05 and |fold change| > 1.2 or <0.83. Enrichment analyses (GO, KEGG, *p* < 0.05) were performed for functional interpretation.

### 2.10 Untargeted metabolomics analysis

Six replicates for each group of biofilm samples were harvested and metabolites were extracted (details in Supplementary Material). Metabolites were identified by spectral matching within 10 ppm tolerance against KEGG, HMDB (Human Metabolome Database), and LIPIDMAPS (Lipid Metabolites and Pathways Strategy) databases. Quality control–based normalization (CV < 30%) was applied prior to statistical analysis. Multivariate analyses included principal component analysis (PCA) and partial least squares discriminant analysis (PLS-DA) via the metaX platform, alongside univariate t-tests to find differential metabolites (VIP > 1, *p* < 0.05, fold change ≥2 or ≤0.5). Differential metabolites were visualized using volcano plots and z-score normalized heatmaps. Pearson correlation and pathway enrichment analysis (x/n > y/N, *p* < 0.05) were performed to explore significant metabolic pathways and network alterations.

### 2.11 Integrated multi-omics analysis

Transcriptomic, proteomic, and metabolomic datasets were integrated through pairwise Pearson correlation (*|*r| > 0.7, *p* < 0.05). Significant features were mapped onto KEGG pathways and enriched based on hypergeometric testing (*p* < 0.05).

### 2.12 Statistical analysis

Analysis was conducted using the GraphPad Software (San Diego, USA). Comparison of continuous outcomes between two groups was based on Student’s two-samp*l*e t-test. Comparison of multiple groups was based on the analysis of variance (ANOVA)*. Post-h*oc comparisons after ANOVA were based on Dunnett’s multiple comparison procedure. Statistical significance was set at the 0.05 level.

## 3 Results

### 3.1 Antimicrobial effects of fluoride and EGCG

The inhibitory activity of fluoride and EGCG against *S. mutans* biofilms was first evaluated using crystal violet staining. Both agents exhibited concentration-dependent suppression of biomass (Figures 1A,B). Fluoride produced pronounced inhibition at 250–500 ppm, while concentrations ≤62.5 ppm had negligible effects. EGCG nearly abolished biofilm formation at 2 mg/mL and showed a partial inhibitory effect at 1 mg/mL, whereas lower concentrations (≤0.5 mg/mL) resulted in minimal reduction of biomass, consistent with previous reports (Aragao et al., 2022; Han et al., 2021; Xu et al., 2011; Schneider-Rayman et al., 2021; Hairul Islam et al., 2020).

The checkerboard microdilution assay was subsequently employed to assess combined effects. A clear synergistic interaction was observed between fluoride and EGCG (Figures 1C,D). Notably, the combination of 62.5 ppm fluoride with 0.5 mg/mL EGCG yielded >90% inhibition and an FIC index of 0.5, confirming a synergistic relationship. Increasing either compound above this threshold did not further enhance inhibition, indicating a plateau effect. These findings guided the selection of two conditions for mechanistic studies: High-concentration (HC): 62.5 ppm fluoride + 0.5 mg/mL EGCG, Low-concentration (LC): 15.6 ppm fluoride + 0.125 mg/mL EGCG.

### 3.2 Effects of fluoride and EGCG at different concentrations on growth of *S. mutans*, morphology of biofilms and EPS production

**Figure 2:**
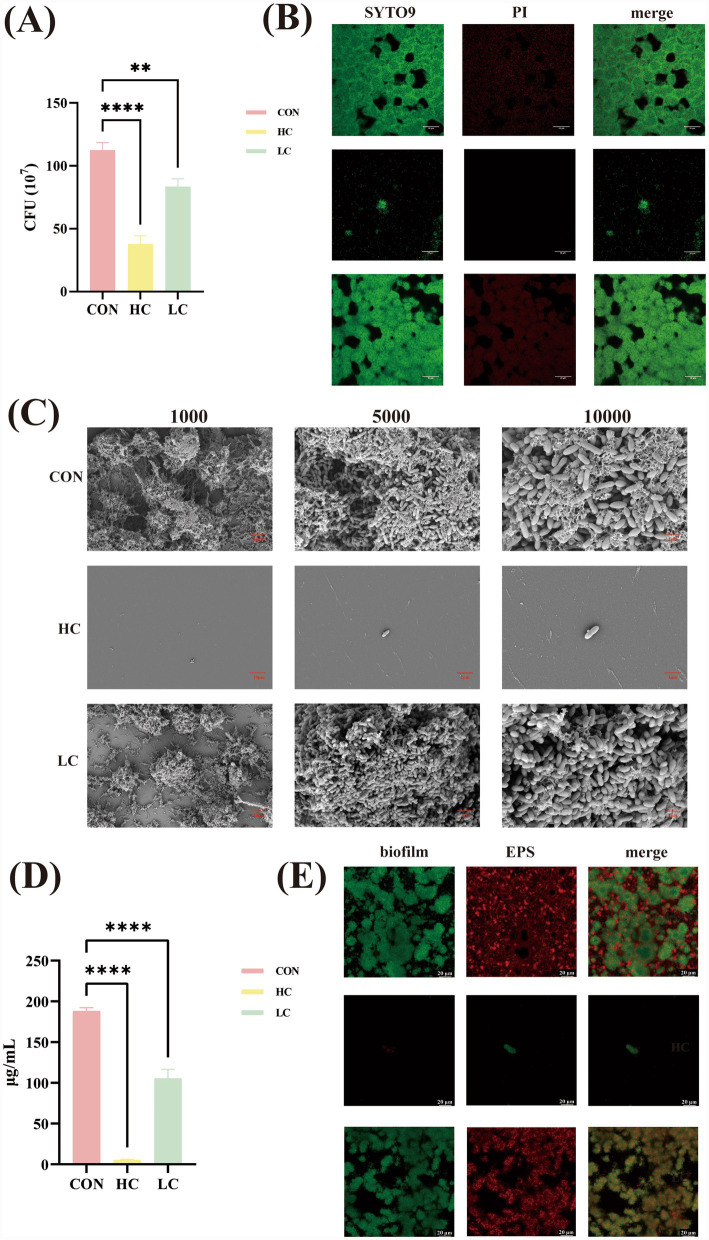
**(A)** CFU counts of different groups. * Represents significant differences between groups (*P <* 0.05). Data are mean ± standard deviations from three independent experiments. **(B)** Biofilms were scanned via confocal laser scanning, where green fluorescence represents live cells, and red fluorescence represents dead cells. **(C)** Biofilms images were got via scanning electron microscope at 1000×, 5,000×, and 10,000 × magnification. **(D)** Quantitative determination of water-insoluble extracellular polysaccharides was measured by the anthrone method. * Represents significant differences between groups (*p <* 0.05). **(E)** Biofilms and EPS were scanned via confocal laser scanning, where green fluorescence represents biofilms, and red fluorescence represents EPS.

To further characterize the biological response to the combined treatments, planktonic growth, biofilm architecture, and EPS formation were examined. CFU enumeration revealed significant differences among the control, LC, and HC groups, with marked viability loss under HC conditions (Figure 2A).

CLSM analysis demonstrated substantial structural disruption in HC-treated biofilms (Figure 2B). Biofilms in the control group displayed dense, multilayered microcolonies, whereas HC treatment resulted in sparse, disorganized bacterial clusters with visibly reduced biomass. The LC group exhibited only minor structural alterations.

SEM imaging corroborated these observations, with untreated biofilms showing extensive extracellular matrix and tightly packed cells (Figure 2C). HC treatment nearly eliminated biofilm formation, leaving only scattered bacterial remnants and markedly diminished matrix material. LC-treated biofilms more closely resembled controls, retaining aggregated structures and intact cellular morphology. Importantly, no overt signs of bacterial lysis were detected in any group.

Consistent with these structural disruptions, HC treatment significantly reduced water-insoluble EPS as quantified by the anthrone assay (Figure 2D). CLSM visualization of EPS further confirmed these findings, showing a near-complete absence of Concanavalin A–stained EPS in the HC group (Figure 2E). Together, these results demonstrated that fluoride and EGCG acted cooperatively to impair *S. mutans* viability, disrupt biofilm organization, and suppress EPS production.

### 3.3 Transcriptome analysis

**Figure 3:**
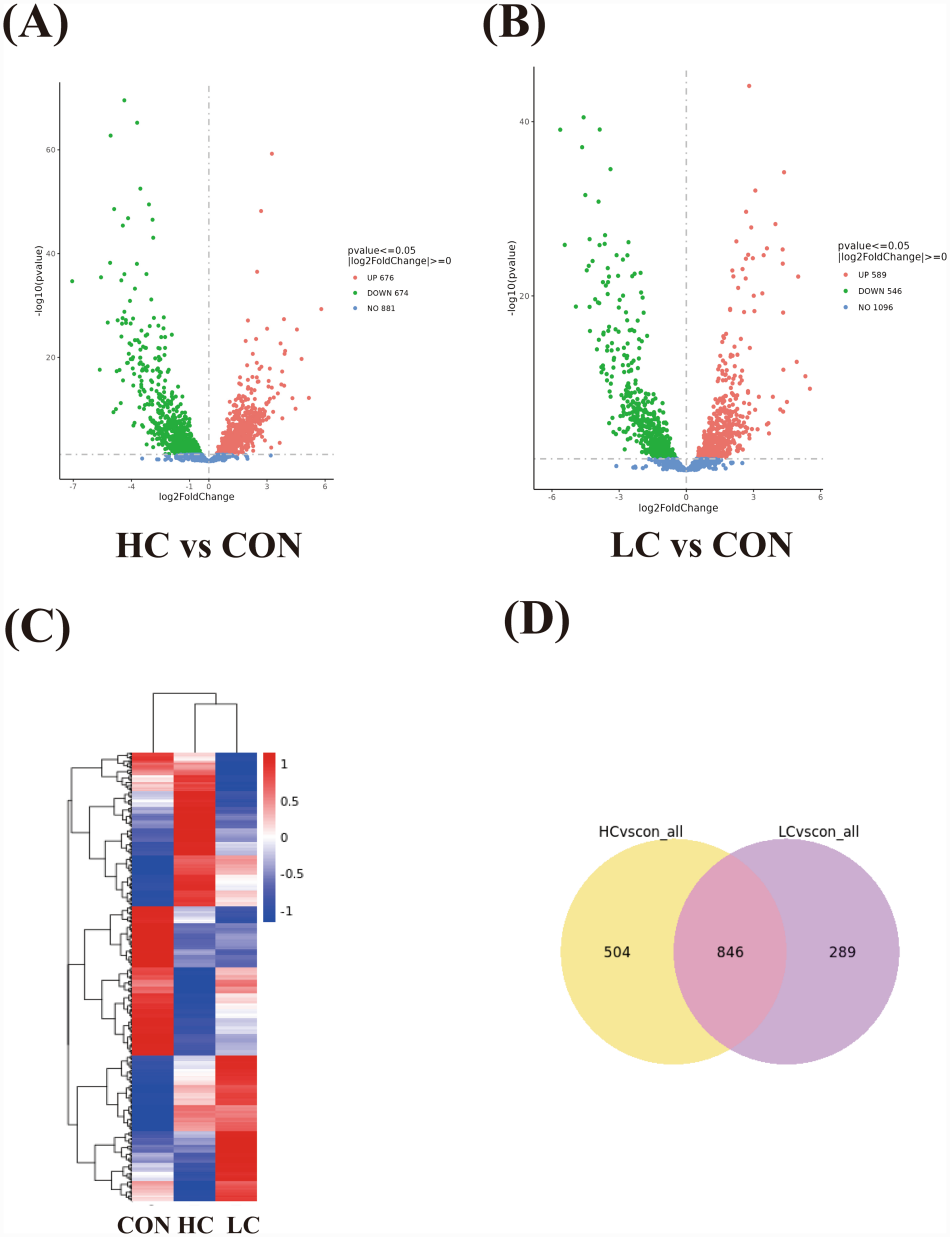
Transcriptome analysis between different groups. **(A,B)** Volcano plot showed differentially expressed genes of HC vs. control comparison and LC vs. control comparison. **(C)** Heat map of control, HC, and LC groups. The red and blue columns represent upregulation and downregulation, respectively. **(D)** 846 overlapping differential expressed genes were showed between HC vs. control comparison and LC vs. control comparison, while 504 genes only expressed in HC vs. control comparison, and 289 genes only expressed in LC vs. control comparison.

To elucidate molecular responses underlying these phenotypic changes, transcriptomic profiling was conducted. Differential expression analysis revealed extensive transcriptional remodeling in both treatment groups. In the HC vs. control comparison, 1,350 genes were differentially expressed (674 downregulated, 676 upregulated, Figures 3A,B). The LC vs. control comparison yielded 1,135 DEGs, including 546 downregulated and 589 upregulated genes. The heat map distinctively showed the transcriptional response of fluoride and EGCG (Figure 3C). In Venn diagram analysis, 846 DEGs were identified between the two comparisons (Figure 3D), indicating substantial overlap in transcriptional responses.

GO enrichment summarized the top 30 terms in both HC vs. control and LC vs. control comparisons (Supplementary Tables S1, S2, Supplementary Figures S1A,B). One significant GO term oxidation–reduction processes, which is relative to carbohydrate metabolism was found in LC vs. control, while no terms were significant in HC vs. control (Table 1). KEGG pathway analysis (Supplementary Figures S1C,D) revealed butanoate metabolism (smu00650) significantly enriched in HC vs. control comparison, a pathway implicated in metabolic flexibility, redox balance, and the provision of acetyl-CoA for EPS biosynthesis. Within this pathway, 13 genes up-regulated and 2 were down-regulated (Table 2). No significant pathways were observed in LC vs. control comparison.

**Table 1:** Significant GO term in LC vs. control comparison.

Category	GOID	Description	GeneRatio	BgRatio	*P*-value	padj
BP	GO:0055114	Oxidation–reduction process	48/389	65/785	0.0000	0.0060

**Table 2:** Significant KEGG term in HC vs. control comparison.

KEGGID	Description	Gene ratio	Bg ratio	*P*-value	padj	Count	Up	Down
smu00650	Butanoate metabolism	15/517	15/828	0.0008	0.0467	15	13	2

### 3.4 TMT labeling proteomic analysis

**Figure 4:**
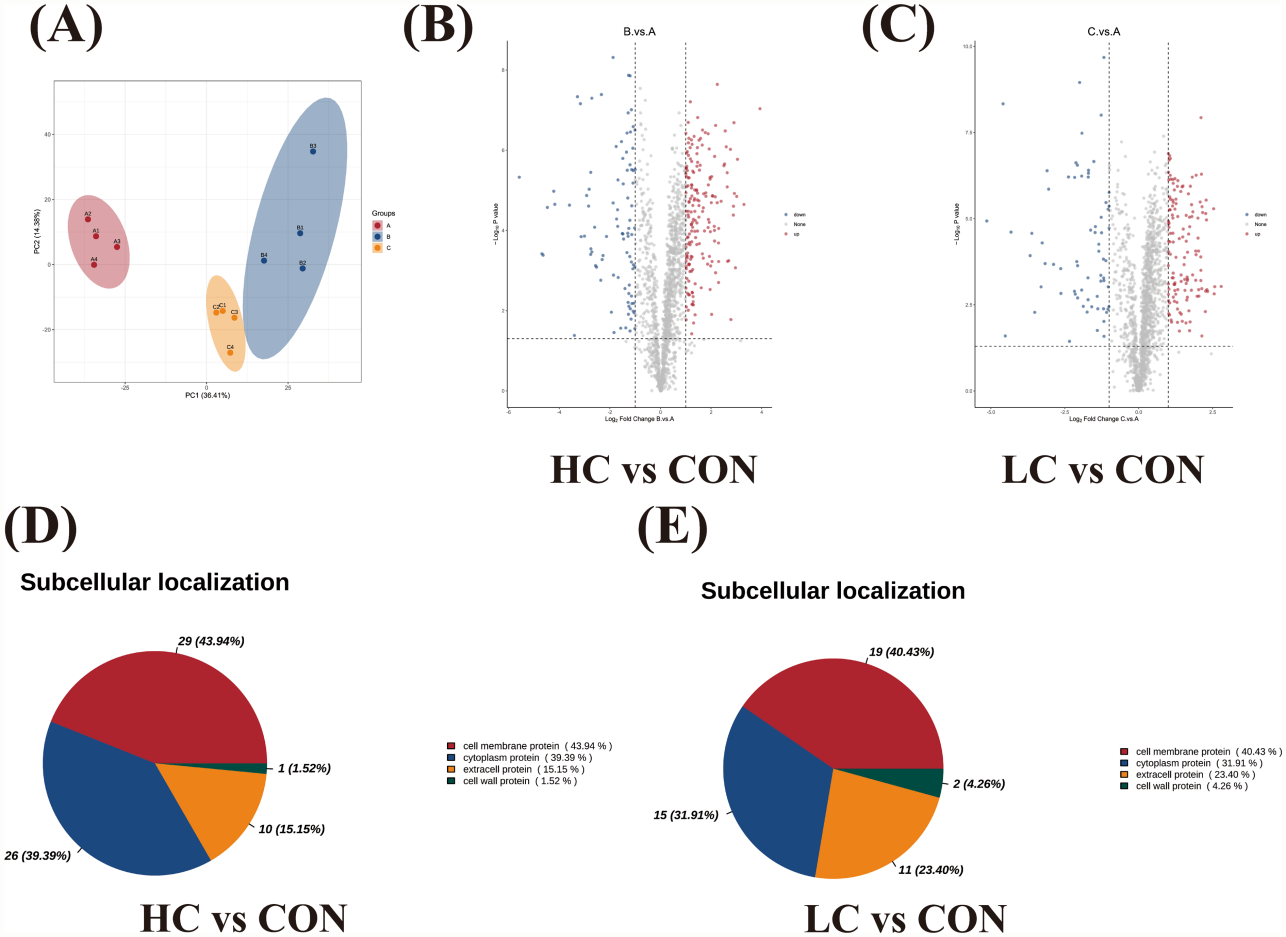
TMT labeling proteomic analysis. **(A)** Unsupervised principal component analysis (PCA) of **(A)** control group, **(B)** HC group, **(C)** LC group. **(B,C)** Volcano plot showed differentially expressed proteins of HC vs. control comparison and LC vs. control comparison. **(D,E)** Proportional subcellular distribution of DEPs were showed, and most kinds of DEPs were located as nucleus proteins in both groups.

To complement transcriptomic data and identify protein-level responses, TMT-based quantitative proteomics was performed. Principal component analysis revealed partial separation of control, LC and HC groups along PC1 (36.41% of variance) and PC2 (14.38%), although substantial overlap remained (Figure 4A), suggesting moderate but consistent proteomic reprogramming.

In total, 1,628 proteins were detected in HC vs. control comparison, with 181 significantly expressed upregulated and 95 downregulated proteins (Figure 4B). In LC vs. control comparison, 1,632 proteins were identified, including 134 significantly upregulated and 69 downregulated proteins (Figure 4C). Significant GO terms and KEGG terms were presented (Supplementary Figures S2A–D, Supplementary Tables S1–S4). It could be visualized that most DEPs were localized to the cell membrane protein in both comparisons (Figures 4D,E). Integration of DEPs with GO and KEGG enrichment analyses identified 8 proteins in HC vs. control comparison that were significantly enriched in both annotation systems (Table 3). These DEPs were classified into four functional categories: extracellular polysaccharide synthesis–related proteins, nutrient sensing and regulatory proteins, metabolic enzymes, and stress response–associated proteins. Proteins involved in extracellular polysaccharide synthesis were predominantly downregulated. Three glucosyltransferases (AAN58619.1, AAN58705.1, AAN58706.1) showed significant reduction in abundance and were annotated to glucan biosynthetic processes and glycosyltransferase activity. Levansucrase (AAN59631.1), a *β*-D-fructosyltransferase, was also markedly downregulated. A nutrient-sensing regulatory protein, nitrogen regulatory protein PII (AAN59296.1), annotated to cellular responses to nutrient availability, was significantly downregulated and mapped to the two-component system pathway. Two DEPs were classified as metabolic enzymes. Phosphoribosylaminoimidazolecarboxamide formyltransferase/IMP cyclohydrolase (AAN57826.1), involved in the one-carbon pool by folate pathway, exhibited a significant reduction in abundance. A conserved hypothetical protein (AAN57803.1) was also markedly downregulated and annotated to β-lactam–related catalytic activity and the two-component system. In addition, one stress response–associated protein with antioxidant activity (putative manganese-type superoxide dismutase, FeMnSOD, AAN58363.1) was significantly downregulated. In LC vs. control comparison (Table 4), DEPs including AAN58619.1, AAN58705.1, AAN58706.1, AAN59631.1, AAN59296.1, AAN58363.1 and AAN57803.1 were consistently downregulated and enriched, whereas a putative CitG protein (AAN58712.1), annotated with phosphotransferase activity for substituted phosphate groups, was significantly upregulated.

**Table 3:** Proteins with significant GO and KEGG enrichment in HC vs. control comparison.

Protein ID	Protein description	Regulation	FC	*P*-value	KEGG annotations	GO annotations
AAN57803.1	Conserved hypothetical protein	Down	0.15	0.0004	map02020 Two-component system	BP:beta-lactam antibiotic catabolic process; MF:beta-lactamase activity
AAN57826.1	Putative phosphoribosylaminoimidazolecarboxamide formyltransferase_IMP cyclohydrolase	Down	0.47	0.0000	map00670 One carbon pool by folate	MF:transferase activity, transferring one-carbon groups
AAN58363.1	Putative manganese_type superoxide dismutase, Fe_Mn_SOD	Down	0.36	0.0001	map04146 Peroxisome	MF:antioxidant activity
AAN58619.1	glucosyltransferase_S	Down	0.11	0.0001	map02020 Two-component system	BP:glucan biosynthetic process; MF:glucosyltransferase activity; MF:hexosyltransferase activity
AAN58705.1	glucosyltransferase_I	Down	0.14	0.0000	map02020 Two-component system	BP:glucan biosynthetic process; MF:glucosyltransferase activity; MF:hexosyltransferase activity
AAN58706.1	glucosyltransferase_SI	Down	0.45	0.0043	map02020 Two-component system	BP:glucan biosynthetic process; MF:glucosyltransferase activity; MF:hexosyltransferase activity
AAN59296.1	Putative nitrogen regulatory protein PII	Down	0.27	0.0000	map02020 Two-component system	BP:response to nutrient levels
AAN59631.1	levansucrase precursor_ beta_D_fructosyltransferase	Down	0.04	0.0004	map02020 Two-component system	BP:response to nutrient levels; MF:hexosyltransferase activity

**Table 4:** Proteins with significant GO and KEGG enrichment in LC vs. control comparison.

Protein ID	Protein description	Regulation	FC	*P*-value	KEGG annotations	GO annotations
AAN57803.1	Conserved hypothetical protein [*Streptococcus mutans* UA159]	Down	0.12	0.0000	map02020 Two-component system	BP:beta-lactam antibiotic catabolic process; MF:beta-lactamase activity; BP:catabolic process
AAN58363.1	putative manganese_type superoxide dismutase, Fe_Mn_SOD [*Streptococcus mutans* UA159]	Down	0.46	0.0002	map04146 Peroxisome	MF:antioxidant activity
AAN58619.1	glucosyltransferase_S [*Streptococcus mutans* UA159]	Down	0.14	0.0002	map00500 Starch and sucrose metabolism; map02020 Two-component system	BP:glucan biosynthetic process; MF:glucosyltransferase activity; MF:hexosyltransferase activity
AAN58705.1	glucosyltransferase_I [*Streptococcus mutans* UA159]	Down	0.49	0.0042	map00500 Starch and sucrose metabolism; map02020 Two-component system	BP:glucan biosynthetic process; MF:glucosyltransferase activity; MF:hexosyltransferase activity
AAN58706.1	glucosyltransferase_SI [*Streptococcus mutans* UA159]	Down	0.44	0.0041	map00500 Starch and sucrose metabolism; map02020 Two-component system	BP:glucan biosynthetic process; MF:glucosyltransferase activity; MF:hexosyltransferase activity
AAN58712.1	putative CitG protein [*Streptococcus mutans* UA159]	Up	2.01	0.0024	map02020 Two-component system	MF:phosphotransferase activity, for other substituted phosphate groups
AAN59296.1	putative nitrogen regulatory protein PII [*Streptococcus mutans* UA159]	Down	0.49	0.0000	map02020 Two-component system	BP:response to nutrient levels
AAN59631.1	levansucrase precursor_ beta_D_fructosyltransferase [*Streptococcus mutans* UA159]	Down	0.10	0.0001	map00500 Starch and sucrose metabolism; map02020 Two-component system	MF:hexosyltransferase activity; BP:response to nutrient levels

### 3.5 Untargeted metabolomics analysis

**Figure 5:**
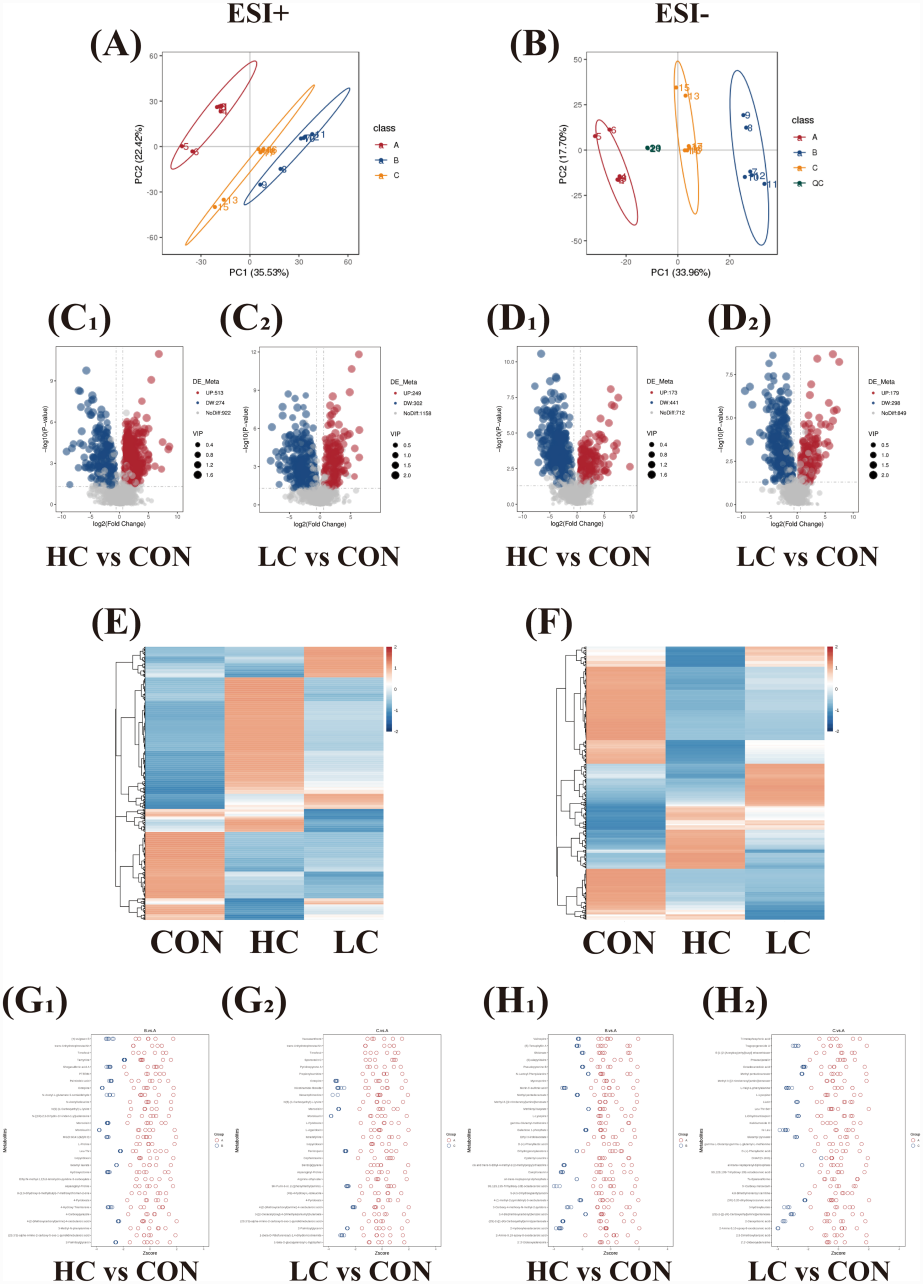
Untargeted metabolomics analysis. Positive mode: **(A, C**_**1**_**, C**_**2**_**, E, G**_**1**_**, G**_**2**_**, H**_**1**_**, H**_**2**_**)**. Negative mode: **(B, D1, D2, F, H1, H2)**. **(A,B)** Unsupervised principal component analysis (PCA) of **(A)** control group, **(B)** HC group, **(C)** LC group. **(C**_**1**_**, C**_**2**_**, D**_**1**_**, D**_**2**_**)** Volcano plot showed different metabolites of HC vs. control comparison and LC vs. control comparison. **(E,F)** Heat map of control, HC, and LC groups. The red and blue columns represent upregulation and downregulation, respectively. **(G**_**1**_**, G**_**2**_**, H**_**1**_**, H**_**2**_**)** *Z*-score plot of metabolic profile divergence.

Untargeted metabolomics was performed to capture functional metabolic changes underlying transcriptomic and proteomic responses. PCA confirmed stable data quality (Figures 5A,B). In HC vs. control comparison, treating with fluoride and EGCG significantly modulated the production of metabolites (Figures 5C_1_, D_1_) with 513 metabolites up-regulated at positive mode (ESI^+^, Electrospray Ionization), and 274 metabolites down-regulated at negative mode (ESI^−^). The clustering heatmap and Z-score plot distinctly illustrated the metabolic profile divergence between treatment groups exposed to high and low concentrations of fluoride and EGCG when compared with control group (Figures 5E, G_1_, H_1_). In LC vs. control comparison, 249 metabolites were up-regulated at positive mode and 302 metabolites were down-regulated at negative mode (Figures 5C_2_, D_2_). The clustering heatmap was also shown (Figures 5F,G_2_, H_2_).

Annotation of differential metabolites followed by pathway mapping and in-depth analysis enabled the identification of key metabolic pathways closely associated with these metabolites. In HC vs. control comparison, metabolomic interrogation disclosed pronounced rearrangements within 20 key metabolites and three biosynthetic super-pathways (Table 5), containing amino-acid biosynthesis with Ribose-5-phosphate, dihydroxyacetone phosphate, l-glutamic acid 5-phosphate, alpha-isopropylmalate, N-acetyl-l-glutamate, L-2-aminoadipic acid, Sedoheptulose 7-phosphate and O-succinylhomoserine, tyrosine catabolism with homogentisic acid, 4-hydroxyphenylpyruvic acid, 2,5-dihydroxybenzaldehyde, 3-methoxytyramine, succinic acid, indole-5,6-quinone and 4-hydroxycinnamic acid and aromatic amino-acid biosynthesis with shikimate, phosphoenolpyruvate, fosfructose, d-erythrose 4-phosphate and phenylpyruvate. In LC vs. control comparison, 12 key metabolites and three core KEGG pathways were listed (Table 6). Chemical carcinogenesis was populated by a five-member signature comprising the polycyclic aromatic hydrocarbon benzo[a]pyrene, the heterocyclic aromatic amine 2-amino-1-methyl-6-phenyl-imidazo[4,5-b]pyridine (PhIP), the nitrosoarene 4-nitrosobiphenyl, the tobacco-specific nitrosamine 4-(methylnitrosamino)-1-(3-pyridyl-N-oxide)-butan-1-ol, and the genotoxic N-acetoxy-MeIQx. The phosphonate/phosphinate module exhibited elevated phosphoenolpyruvate, *α*-D-ribose-5-phosphate, D-ribose-1,5-bisphosphate and phosphocholine. Within the vitamin B6 network, isopyridoxal, pyridoxamine and 4-pyridoxate were selectively enriched, reflecting an adaptive acceleration of pyridoxal-phosphate-dependent transamination reactions.

**Table 5:** Metabolites with KEGG pathways in HC vs. control comparison.

Ion Mode	Compound_ID	Name	Up/Down	Significant KEGG pathways	*P*-value
Positive	Com_252_pos	4-Hydroxyphenylpyruvic acid	Up	Tyrosine metabolism	0.0000
Positive	Com_34_pos	2,5-Dihydroxybenzaldehyde	Up	Tyrosine metabolism	0.0000
Positive	Com_192_pos	Homogentisic acid	Up	Tyrosine metabolism	0.0000
Positive	Com_189_pos	3-Methoxytyramine	Up	Tyrosine metabolism	0.0004
Positive	Com_1359_pos	SUCCINIC ACID	Up	Tyrosine metabolism	0.0004
Positive	Com_1616_pos	Indole-5,6-quinone	Up	Tyrosine metabolism	0.0119
Positive	Com_170_pos	4-Hydroxycinnamic acid	Down	Tyrosine metabolism	0.0000
Negative	Com_78_neg	Phosphoenolpyruvic acid	Up	Phenylalanine, tyrosine and tryptophan biosynthesis; Biosynthesis of amino acids	0.0004
Negative	Com_115_neg	alpha-Isopropylmalate	Up	Biosynthesis of amino acids	0.0016
Negative	Com_162_neg	N-Acetyl-L-glutamic acid	Up	Biosynthesis of amino acids	0.0029
Negative	Com_979_neg	Shikimate	Down	Phenylalanine, tyrosine and tryptophan biosynthesis; Biosynthesis of amino acids	0.0000
Negative	Com_199_neg	O-Succinyhomoserine	Down	Biosynthesis of amino acids	0.0000
Negative	Com_663_neg	Fosfructose	Down	Phenylalanine, tyrosine, and tryptophan biosynthesis	0.0000
Negative	Com_188_neg	D-Erythrose 4-phosphate	Down	Phenylalanine, tyrosine and tryptophan biosynthesis; Biosynthesis of amino acids	0.0000
Negative	Com_1404_neg	RIBOSE 5-PHOSPHATE	Down	Biosynthesis of amino acids	0.0000
Negative	Com_947_neg	Dihydroxyacetone phosphate	Down	Biosynthesis of amino acids	0.0003
Negative	Com_1364_neg	L-Glutamic acid 5-phosphate	Down	Biosynthesis of amino acids	0.0007
Negative	Com_60_neg	Phenylpyruvic acid	Down	Phenylalanine, tyrosine, and tryptophan biosynthesis; Biosynthesis of amino acids	0.0011
Negative	Com_893_neg	L-2-Aminoadipic acid	Down	Biosynthesis of amino acids	0.0035
Negative	Com_2092_neg	Sedoheptulose 7-phosphate	Down	Biosynthesis of amino acids	0.0051

**Table 6:** Metabolites with KEGG pathways in LC vs. control comparison.

Ion mode	Compound_ID	Name	Up/Down	Significant KEGG pathways	*P*-value
Positive	Com_300_pos	Isopyridoxal	Up	Vitamin B6 metabolism	0.0000
Positive	Com_2060_pos	4-Pyridoxate	Down	Vitamin B6 metabolism	0.0000
Positive	Com_745_pos	Benzo[a]pyrene	Up	Chemical carcinogenesis	0.0000
Positive	Com_713_pos	PhIP	Up	Chemical carcinogenesis	0.0003
Positive	Com_1898_pos	Pyridoxamine	Up	Vitamin B6 metabolism	0.0020
Positive	Com_2480_pos	4-(Methylnitrosamino)-1-(3-pyridyl-N-oxide)-1-butanol	Up	Chemical carcinogenesis	0.0030
Positive	Com_3061_pos	N-Acetoxy-MeIQx	Up	Chemical carcinogenesis	0.0078
Positive	Com_2062_pos	4-Nitrosobiphenyl	Down	Chemical carcinogenesis	0.0202
Negative	Com_1404_neg	RIBOSE 5-PHOSPHATE	Down	Phosphonate and phosphinate metabolism	0.0000
Negative	Com_2301_neg	D-Ribose 1,5-bisphosphate	Down	Phosphonate and phosphinate metabolism	0.0000
Negative	Com_78_neg	Phosphoenolpyruvic acid	Up	Phosphonate and phosphinate metabolism	0.0000
Negative	Com_779_neg	Foscarnet	Up	Phosphonate and phosphinate metabolism	0.0101

Additionally, 25 overlapped pathways were crossed based on HC vs. control group and LC vs. control group, and the same metabolites were also listed (Supplementary Table S5).

### 3.6 Multi-omics analyses

**Figure 6:**
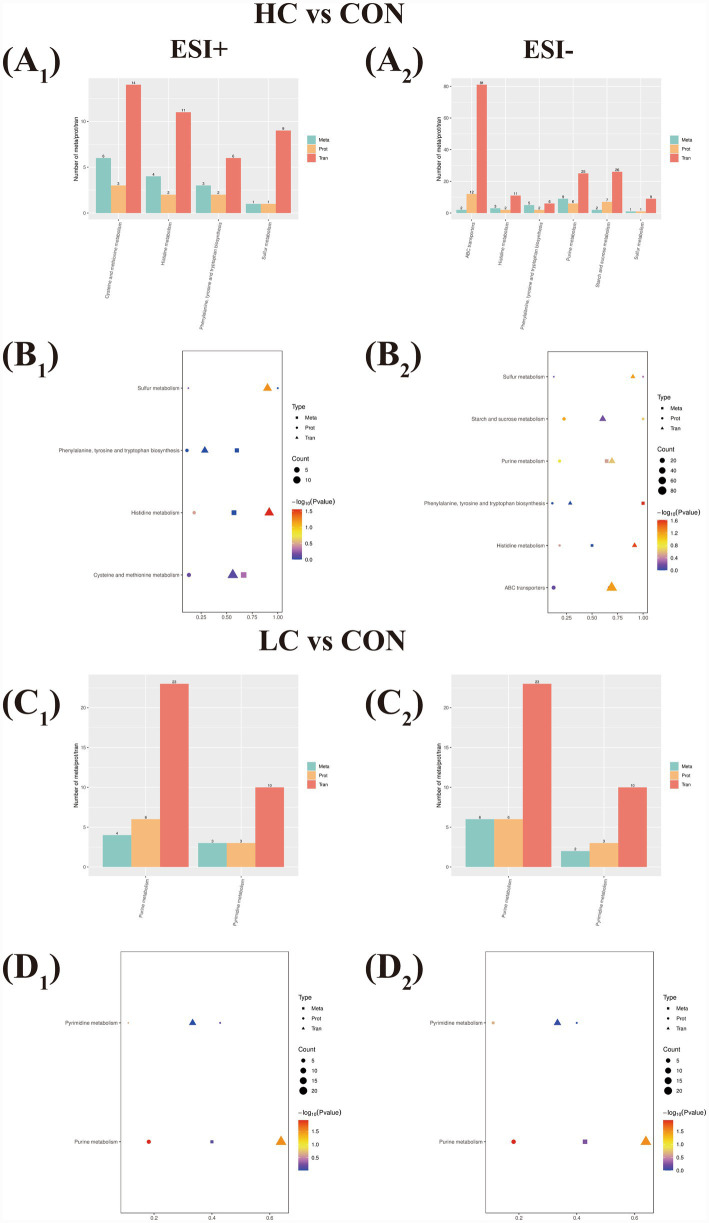
Multi-omics analysis. Positive mode **(A1−D1)**. Negative mode **(A2−D2)**. **(A1, A2)** Crossed KEGG pathways based on transcriptomic, proteomic, and metabolic analysis in HC vs. control comparison. **(B**_**1**_**, B**_**2**_**)** Bubble plot of KEGG enrichment analysis in HC vs. control comparison, and *p <* 0.05 was regarded as significant different pathways. **(C**_**1**_**, C**_**2**_**)** Crossed KEGG pathways based on transcriptomic, proteomic, and metabolic analysis in LC vs. control comparison. **(D**_**1**_**, D**_**2**_**)** Bubble plot of KEGG enrichment analysis in LC vs. control comparison, and *p <* 0.05 was regarded as significant different pathways.

To characterize the molecular alterations induced by different concentration treatments, integrated transcriptomic, proteomic and metabolomic analyses were performed, with metabolomic data acquired in both negative and positive ionization modes. In HC vs. control comparison, amino acid- and carbohydrate metabolism- related pathways were found as the most pronounced pathways. Notably, at both positive and negative modes, histidine metabolism was significantly enriched at the transcriptional level (*p* < 0.05), whereas corresponding proteomic and metabolomic changes were less pronounced. Moreover, at negative mode, phenylalanine, tyrosine and tryptophan biosynthesis showed significant enrichment at the metabolomic level (*p* < 0.05) (Table 7). Several additional pathways were detected across multiple omics layers but did not reach statistical significance (Supplementary Table S6, Figures 6A_1_, A_2_, B_1_, B_2_). For LC vs. control comparison, joint pathway enrichment analyses identified that purine metabolism was the most frequently represented pathway across omics layers at positive and negative modes (Table 8). Notably, purine metabolism exhibited significant enrichment at the transcriptional and proteomic level (*p* < 0.05), whereas corresponding metabolomic changes were less pronounced. Additional enriched pathways above the significance threshold were detected and shown (Supplementary Table S7, Figures 6C_1_, C_2_, D_1_, D_2_).

**Table 7:** Multi-omics analysis in HC vs. control comparison.

Ion mode	Description	*P*-value	Ratio	Count	Type	ID	KO
Positive	Histidine metabolism	0.0280	0.92	11	Tran	SMU_RS05855/SMU_RS05830/SMU_RS05825/SMU_RS05845/SMU_RS05865/SMU_RS05860/SMU_RS00890/SMU_RS05835/SMU_RS05870/SMU_RS05820/SMU_RS05810	smu00340
Positive	Histidine metabolism	0.3398	0.18	2	Prot	AAN58946.1, AAN58948.1	map00340
Positive	Histidine metabolism	1.0000	0.57	4	Meta	trans-urocanate; L-Glutamic acid; 1-Methylhistidine; L-Histidine trimethylbetaine	map00340
Negative	Phenylalanine, tyrosine, and tryptophan biosynthesis	0.9997	0.29	6	Tran	SMU_RS03625/SMU_RS03630/SMU_RS03615/SMU_RS05870/SMU_RS03645/SMU_RS05940	smu00400
Negative	Phenylalanine, tyrosine, and tryptophan biosynthesis	1.0000	0.11	2	Prot	AAN58500.1, AAN59459.1	map00400
Negative	Phenylalanine, tyrosine, and tryptophan biosynthesis	0.0244	1.00	5	Meta	D-Erythrose 4-phosphate; Phenylpyruvic acid; Fosfructose; Phosphoenolpyruvic acid; Shikimate	map00400
Negative	Histidine metabolism	0.0280	0.92	11	Tran	SMU_RS05855/SMU_RS05830/SMU_RS05825/SMU_RS05845/SMU_RS05865/SMU_RS05860/SMU_RS00890/SMU_RS05835/SMU_RS05870/SMU_RS05820/SMU_RS05810	smu00340
Negative	Histidine metabolism	0.3398	0.18	2	Prot	AAN58946.1, AAN58948.1	map00340
Negative	Histidine metabolism	1.0000	0.50	3	Meta	N-Carbamylglutamate; N-Formyl-L-aspartate; hydantoin-5-propionate	map00340

**Table 8:** Multi-omics analysis in LC vs. control comparison.

Ion mode	Description	*P*-value	Ratio	Count	Type	ID	KO
Positive	Purine metabolism	0.0311	0.64	23	Tran	SMU_RS03160/SMU_RS00325/SMU_RS03165/SMU_RS00365/SMU_RS04965/SMU_RS00320/SMU_RS00240/SMU_RS04820/SMU_RS07805/SMU_RS09345/SMU_RS00230/SMU_RS00255/SMU_RS00265/SMU_RS00250/SMU_RS06660/SMU_RS00225/Novel00183/Novel00006/SMU_RS09845/SMU_RS00060/SMU_RS05960/Novel00010/SMU_RS00310	smu00230
Positive	Purine metabolism	0.0121	0.18	6	Prot	AAN57818.1, AAN57819.1, AAN57823.1, AAN57824.1, AAN57837.1, AAN59670.1	map00230
Positive	Purine metabolism	0.7308	0.40	4	Meta	5-Hydroxyisourate; 2’-Deoxyadenosine; Adenosine; Guanosine	map00230
Negative	Purine metabolism	0.0311	0.64	23	Tran	SMU_RS03160/SMU_RS00325/SMU_RS03165/SMU_RS00365/SMU_RS04965/SMU_RS00320/SMU_RS00240/SMU_RS04820/SMU_RS07805/SMU_RS09345/SMU_RS00230/SMU_RS00255/SMU_RS00265/SMU_RS00250/SMU_RS06660/SMU_RS00225/Novel00183/Novel00006/SMU_RS09845/SMU_RS00060/SMU_RS05960/Novel00010/SMU_RS00310	smu00230
Negative	Purine metabolism	0.0121	0.18	6	Prot	AAN57818.1, AAN57819.1, AAN57823.1, AAN57824.1, AAN57837.1, AAN59670.1	map00230
Negative	Purine metabolism	0.5610	0.43	6	Meta	RIBOSE 5-PHOSPHATE; 5-Amino-1-(5-phospho-D-ribosyl)imidazole-4-carboxylic acid; Adenylosuccinic acid; inosine; Inosinic acid; Guanosine 5′-diphosphate	map00230

## 4 Discussion

Fluoride and tea polyphenols remain widely used antimicrobial agents in oral health care, and their combined use has attracted increasing interest as a strategy for enhancing anticaries efficacy. In the present study, we identified a strong synergistic inhibitory effect of fluoride and EGCG on *S. mutans* biofilms, supporting their potential integration into future clinical formulations. Although high concentrations of tea polyphenols (>5 mg/mL) have been reported to exert bactericidal activity, lower doses are generally preferred for safety and translational feasibility (Hirasawa et al., 2006). Consistent with previous findings, EGCG at 0.5–2 mg/mL effectively reduced biofilm formation. Notably, the combination of 62.5 ppm fluoride and 0.5 mg/mL EGCG achieved substantial biofilm inhibition at a relatively low dosage, supporting its potential for effective biofilm control without apparent additional cytotoxic burden (Aragao et al., 2022; Han et al., 2021; Schneider-Rayman et al., 2021). The lack of a strict dose–response relationship, together with maintained efficacy at lower concentrations, highlights the clinical relevance of using optimized low-dose combinations. Accordingly, we defined this combination as the high-concentration (HC) group for mechanistic analyses, while the lowest dose producing minimal biofilm disruption served as the low-concentration (LC) group.

A primary mechanistic insight from this study is the reduction of glucan production through suppression of glucosyltransferases (Gtfs), verified by quantification of water-insoluble EPS. EPS is fundamental to the structural and functional resilience of *S. mutans* biofilms, providing mechanical stability, facilitating bacterial accumulation, forming acidic diffusion gradients, and protecting biofilm-embedded cells from antimicrobial and host-derived challenges (Bowen and Koo, 2011; Koo et al., 2010; Flemming et al., 2023). Disruption of EPS synthesis is thus expected to have profound effects on biofilm maturation and cariogenicity (Koo et al., 2013). Interestingly, however, we found that EPS suppression was not always proportional to biofilm biomass reduction. In the LC group, EPS levels decreased by approximately 50%, whereas the overall biofilm mass showed a less pronounced decline. This divergence suggests that fluoride and EGCG co-treatment alters biofilm physiology via multiple pathways beyond EPS disruption.

To delineate these mechanisms, we employed a comprehensive multi-omics approach. While fluoride and EGCG have individually been studied extensively, their combined effects on global regulatory networks have remained largely underexplored. Our transcriptomic, proteomic, and metabolomic datasets revealed several pathways previously uncharacterized in the context of *S. mutans* biofilm inhibition, thereby broadening the mechanistic landscape underlying the synergistic phenotype.

Transcriptomic analysis showed that redox-associated processes were prominently enriched in the LC group, indicating that low-dose co-treatment may target oxidative stress–responsive regulatory circuits. Redox-sensitive regulatory systems orchestrate the expression of virulence factors including Gtfs, acid tolerance proteins, and EPS synthesis enzymes essential for biofilm robustness (Bitoun and Wen, 2016; Yu et al., 2023). Rex is a transcription factor that regarded as the sensitive regulator of redox systems. It could be activated upon binding to NAD^+^ and inhibited upon association with NADH, thereby modulating the NADH/NAD^+^ ratio and affecting *rex* transcription and *nox* expression (Bitoun and Wen, 2016; Yu et al., 2023; McLaughlin et al., 2010; Pagels et al., 2010; Wang et al., 2008). Spx, another regulatory factor in redox systems, is responsible for most oxidative stress adaptation genes that are related to growth and energy metabolism of *S. mutans* (Baker et al., 2014; Galvao et al., 2015; Kajfasz et al., 2015; Crepps et al., 2016). Other transcription factors like SloR, two-component systems, Cid/Lrg system are also vital for *S. mutans* involving in adapting to the oxidative stress (Yu et al., 2023; De Furio et al., 2017; Rice et al., 2017). These findings suggest that redox imbalance contributes to the observed inhibitory effects, particularly at lower concentrations in which EPS suppression alone cannot fully account for the reduced biofilm formation.

Proteomic analyses complemented these observations by identifying biologically relevant changes at the protein level. A dominant feature of the proteomic profile was the concerted downregulation of enzymes involved in extracellular polysaccharide synthesis, including multiple glucosyltransferases and levansucrase, which are central to glucan and fructan production and biofilm matrix assembly, and are key determinants of cariogenic biofilm virulence (Bowen et al., 2018; Fitri et al., 2025). Suppression of these enzymes at the protein level supports impaired EPS formation and reduced biofilm stability. In parallel, key regulatory components associated with nutrient sensing and two-component signal transduction, particularly nitrogen regulatory protein PII, were consistently downregulated, suggesting disruption of environmental responsiveness and signal integration, a phenomenon aligned with complex regulatory control over stress and metabolic adaptation in *S. mutans* proteomes under stress conditions (Tinder et al., 2022). Proteins involved in central metabolism, including a folate-dependent enzyme involved in purine biosynthesis, also showed reduced abundance, indicating metabolic constraint under treatment conditions and limited biosynthetic capacity (Gelinas et al., 2021). Moreover, decreased levels of MnSOD point to weakened oxidative stress defense, a mechanism closely linked to reduced stress tolerance and competitiveness of *S. mutans* within oral biofilms (Yu et al., 2023). A conserved hypothetical protein linked to regulatory and catabolic processes was similarly suppressed, implying broader perturbation of adaptive networks. Notably, selective upregulation of a phosphotransferase-related regulatory protein under LC conditions suggests a partial compensatory response at lower treatment intensity, consistent with recent evidence that exposure to the sublethal dose triggers remodeling of regulatory and signaling pathways (Carvalho et al., 2021). Collectively, these proteomic alterations demonstrate that the treatments attenuate *S. mutans* virulence and persistence by simultaneously targeting extracellular matrix production, regulatory signaling, metabolism and stress tolerance.

Metabolomic profiling provided additional layers of insight by revealing coordinated shifts in core metabolic pathways. In HC group, upregulation of *α*-isopropylmalate, N-acetyl-L-glutamate, and phosphoenolpyruvate alongside decreased ribose-5-phosphate and dihydroxyacetone phosphate suggests redistribution of metabolic flux away from energy-generating pathways toward essential biosynthetic processes. Although such shifts may theoretically sustain growth, our findings indicate that they ultimately fail to support EPS biosynthesis, contributing to impaired biofilm maturation (Seregina et al., 2025; Shi et al., 2015). The pronounced accumulation of homogentisic acid highlights modulation of tyrosine catabolism, though the functional implications of this metabolite in *S. mutans* remain unclear (Lorquin et al., 2022). In LC group, enrichment of benzo[a]pyrene- and PhIP-associated metabolites along with vitamin B6 intermediates suggests alterations in xenobiotic metabolism and B6-dependent reactions, consistent with adaptive responses to environmental stress (Zhang et al., 2019; Mooney et al., 2009).

Integrated multi-omics analyses revealed a clear concentration-dependent molecular response of *S. mutans* to the combined treatment of fluoride and EGCG. Although both low and high concentration conditions elicited substantial transcriptional alterations with considerable overlap, differences became apparent at the proteomic and metabolomic levels. Under low concentration exposure, purine metabolism was consistently detected across transcriptomic and proteomic layers, suggesting coordinated modulation of nucleotide and energy-related processes. Such cross-omics concordance was consistent with previous observations that sublethal antimicrobial stress can induce regulatory remodeling rather than broad metabolic collapse (Andersson and Hughes, 2014; Davies and Davies, 2010). In contrast, high concentration treatment was characterized by reduced cross-omics concordance and predominant downregulation of proteins involved in EPS synthesis, central metabolic pathways and stress response systems. These changes were accompanied by widespread perturbations in amino acid and carbon metabolism at the metabolite level, reflecting a more constrained cellular state. Similar decoupling between transcriptional responses and downstream metabolic outputs under stronger antimicrobial pressure has been reported in multi-omics studies of bacterial stress adaptation (Jensen et al., 2017). Given the established dependence of EPS biosynthesis on nucleotide availability and energy metabolism in *S. mutans* (Bowen and Koo, 2011), the observed metabolic disruptions are likely associated with the impaired biofilm formation detected phenotypically. Together, these findings support a concentration-dependent framework in which fluoride and EGCG exert synergistic inhibitory effects through distinct molecular response profiles, rather than a single uniform mechanism, consistent with prior reports on polyphenol-mediated modulation of bacterial metabolism and stress responses (Daglia, 2012).

This study has several limitations. The absence of single-agent treatment groups in omics analyses limits our ability to attribute specific molecular changes to fluoride or EGCG individually. *In vivo* validation is also necessary to confirm the translational potential of these findings, as dynamic oral environmental factors may modify the effects observed *in vitro*. Furthermore, the concentration-dependent metabolic reprogramming identified here requires clarification through targeted functional assays.

In conclusion, for clinical applications, using the concentration of fluoride at 62.5 ppm and EGCG at 0.5 mg/mL is promising and safe. Our findings demonstrate that fluoride and EGCG synergistically inhibit *S. mutans* biofilm formation through coordinated mechanisms involving EPS suppression, disruption of redox homeostasis, and reprogramming of core metabolic pathways such as amino acid biosynthesis, tyrosine metabolism, and butanoate metabolism. Incorporating EGCG into fluoride-based strategies may broaden the functional capabilities of conventional fluoride therapies while maintaining safety and efficacy, supporting the development of next-generation anticaries agents. Future work should emphasize mechanistic dissection of concentration-dependent effects, incorporation of single-agent controls, and validation *in vivo* to further refine and optimize this synergistic approach.

## Data Availability

The raw RNA sequencing data have been deposited in the NCBI Sequence Read Archive (SRA) under accession number PRJNA1345365. The proteomics data were deposited in the ProteomeXchange Consortium via the PRIDE partner repository under dataset identifier PXD069651. The datasets generated and analysed during the current study are available from the corresponding author on reasonable request.

## References

[ref1] Andersson D. I. Hughes D. (2014). Microbiological effects of sublethal levels of antibiotics. Nat. Rev. Microbiol. 12, 465–478. doi: 10.1038/nrmicro3270, 24861036

[ref2] Aragao M. G. B. Aires C. P. Corona S. A. M. (2022). Effects of the green tea catechin epigallocatechin-3-gallate on *Streptococcus mutans* planktonic cultures and biofilms: systematic literature review of in vitro studies. Biofouling 38, 687–695. doi: 10.1080/08927014.2022.2116320, 36017657

[ref3] Baker J. L. Derr A. M. Karuppaiah K. MacGilvray M. E. Kajfasz J. K. Faustoferri R. C. . (2014). *Streptococcus mutans* NADH oxidase lies at the intersection of overlapping regulons controlled by oxygen and NAD+ levels. J. Bacteriol. 196, 2166–2177. doi: 10.1128/JB.01542-14, 24682329 PMC4054193

[ref4] Bitoun J. P. Wen Z. T. (2016). Transcription factor rex in regulation of pathophysiology in oral pathogens. Mol Oral Microbiol 31, 115–124. doi: 10.1111/omi.12114, 26172563 PMC4713358

[ref5] Bowen W. H. Burne R. A. Wu H. Koo H. (2018). Oral biofilms: pathogens, matrix, and polymicrobial interactions in microenvironments. Trends Microbiol. 26, 229–242. doi: 10.1016/j.tim.2017.09.008, 29097091 PMC5834367

[ref6] Bowen W. H. Koo H. (2011). Biology of *Streptococcus mutans*-derived glucosyltransferases: role in extracellular matrix formation of cariogenic biofilms. Caries Res. 45, 69–86. doi: 10.1159/000324598, 21346355 PMC3068567

[ref7] Buzalaf M. A. R. Pessan J. P. Honorio H. M. Ten Cate J. M. (2011). Mechanisms of action of fluoride for caries control. Monogr. Oral Sci. 22, 97–114. doi: 10.1159/000325151, 21701194

[ref8] Cao Y. Zhou Y. Chen D. Wu R. Guo L. Lin H. (2020). Proteomic and metabolic characterization of membrane vesicles derived from *Streptococcus mutans* at different pH values. Appl. Microbiol. Biotechnol. 104, 9733–9748. doi: 10.1007/s00253-020-10563-6, 33064184

[ref9] Carvalho A. Krin E. Korlowski C. Mazel D. Baharoglu Z. (2021). Interplay between sublethal aminoglycosides and quorum sensing: consequences on survival in *V. cholerae*. Cells 10:3227. doi: 10.3390/cells10113227, 34831448 PMC8621022

[ref10] Chen D. Li J. Pan T. Wu R. Tao Y. Lin H. (2021). The broad-spectrum antibiofilm activity of amyloid-forming hexapeptides. Microb. Biotechnol. 14, 656–667. doi: 10.1111/1751-7915.13721, 33248016 PMC7936291

[ref11] Crepps S. C. Fields E. E. Galan D. Corbett J. P. Von Hasseln E. R. Spatafora G. A. (2016). The SloR metalloregulator is involved in the *Streptococcus mutans* oxidative stress response. Mol Oral Microbiol 31, 526–539. doi: 10.1111/omi.12147, 26577188 PMC4871787

[ref12] Daglia M. (2012). Polyphenols as antimicrobial agents. Curr. Opin. Biotechnol. 23, 174–181. doi: 10.1016/j.copbio.2011.08.007, 21925860

[ref13] Davies J. Davies D. (2010). Origins and evolution of antibiotic resistance. Microbiol. Mol. Biol. Rev. 74, 417–433. doi: 10.1128/MMBR.00016-10, 20805405 PMC2937522

[ref14] De Furio M. Ahn S. J. Burne R. A. Hagen S. J. (2017). Oxidative stressors modify the response of *Streptococcus mutans* to its competence signal peptides. Appl. Environ. Microbiol. 83, e01345–17. doi: 10.1128/AEM.01345-17, 28887419 PMC5666127

[ref15] Dehghani M. Abtahi M. Sadeghian H. Shafaee H. Tanbakuchi B. (2015). Combined chlorhexidine-sodiumfluoride mouthrinse for orthodontic patients: clinical and microbiological study. J Clin Exp Dent 7, e569–e575. doi: 10.4317/jced.51979 26644831 PMC4663057

[ref16] Dzoyem J. P. Tsemeugne J. Pone Kamdem B. Foyou Meupiap R. Kuate B. A. Mkounga P. . (2025). Antibacterial, antibiofilm and anti-quorum sensing activities of 1,2,3,5-tetrazine derivatives linked to a benzothiazole moiety. PLoS One 20:e0318135. doi: 10.1371/journal.pone.0318135, 40460187 PMC12133013

[ref17] Featherstone J. D. (1999). Prevention and reversal of dental caries: role of low level fluoride. Community Dent. Oral Epidemiol. 27, 31–40, 10086924 10.1111/j.1600-0528.1999.tb01989.x

[ref18] Fitri D. K. Tuygunov N. Wan Harun W. H. A. Purwasena I. A. Cahyanto A. Zakaria M. N. (2025). Key virulence genes associated with *Streptococcus mutans* biofilm formation: a systematic review. Front Oral Health. 6:1654428. doi: 10.3389/froh.2025.1654428 40933845 PMC12417473

[ref19] Flemming H. C. van Hullebusch E. D. Neu T. R. Nielsen P. H. Seviour T. Stoodley P. . (2023). The biofilm matrix: multitasking in a shared space. Nat. Rev. Microbiol. 21, 70–86. doi: 10.1038/s41579-022-00791-0, 36127518

[ref20] Frencken J. E. Sharma P. Stenhouse L. Green D. Laverty D. Dietrich T. (2017). Global epidemiology of dental caries and severe periodontitis - a comprehensive review. J. Clin. Periodontol. 44, S94–S105. doi: 10.1111/jcpe.12677 28266116

[ref21] Galvao L. C. Miller J. H. Kajfasz J. K. Scott-Anne K. Freires I. A. Franco G. C. . (2015). Transcriptional and phenotypic characterization of novel Spx-regulated genes in *Streptococcus mutans*. PLoS One 10:e0124969. doi: 10.1371/journal.pone.0124969, 25905865 PMC4408037

[ref22] Gelinas M. Museau L. Milot A. Beauregard P. B. (2021). The de novo purine biosynthesis pathway is the only commonly regulated cellular pathway during biofilm formation in TSB-based medium in *Staphylococcus aureu*s and *Enterococcus faecalis*. Microbiol Spectr. 9:e0080421. doi: 10.1128/Spectrum.00804-21, 34935415 PMC8693917

[ref23] Hairul Islam M. I. Arokiyaraj S. Kuralarasan M. Senthil Kumar V. Harikrishnan P. Saravanan S. . (2020). Inhibitory potential of EGCG on *Streptococcus mutans* biofilm: a new approach to prevent Cariogenesis. Microb. Pathog. 143:104129. doi: 10.1016/j.micpath.2020.104129 32169491

[ref24] Han S. Abiko Y. Washio J. Luo Y. Zhang L. Takahashi N. (2021). Green tea-derived epigallocatechin gallate inhibits acid production and promotes the aggregation of *Streptococcus mutans* and non-mutans streptococci. Caries Res. 55, 205–214. doi: 10.1159/000515814, 34010838

[ref25] Han S. Washio J. Abiko Y. Zhang L. Takahashi N. (2023). Green tea-derived catechins suppress the acid productions of *Streptococcus mutans* and enhance the efficiency of fluoride. Caries Res. 57, 255–264. doi: 10.1159/000534055, 37699359 PMC10641802

[ref26] Hirasawa M. Takada K. Otake S. (2006). Inhibition of acid production in dental plaque bacteria by green tea catechins. Caries Res. 40, 265–270, . doi: 10.1159/000092236 16707877

[ref27] Jensen P. A. Zhu Z. van Opijnen T. (2017). Antibiotics disrupt coordination between transcriptional and phenotypic stress responses in pathogenic bacteria. Cell Rep. 20, 1705–1716. doi: 10.1016/j.celrep.2017.07.062, 28813680 PMC5584877

[ref28] Kajfasz J. K. Rivera-Ramos I. Scott-Anne K. Gregoire S. Abranches J. Lemos J. A. (2015). Transcription of oxidative stress genes is directly activated by SpxA1 and, to a lesser extent, by SpxA2 in *Streptococcus mutans*. J. Bacteriol. 197, 2160–2170. doi: 10.1128/JB.00118-15, 25897032 PMC4455267

[ref29] Kazeminia M. Abdi A. Shohaimi S. Jalali R. Vaisi-Raygani A. Salari N. . (2020). Dental caries in primary and permanent teeth in children's worldwide, 1995 to 2019: a systematic review and meta-analysis. Head Face Med 16:22. doi: 10.1186/s13005-020-00237-z, 33023617 PMC7541284

[ref30] Koo H. Falsetta M. L. Klein M. I. (2013). The exopolysaccharide matrix: a virulence determinant of cariogenic biofilm. J. Dent. Res. 92, 1065–1073. doi: 10.1177/0022034513504218, 24045647 PMC3834652

[ref31] Koo H. Sheng J. Nguyen P. T. Marquis R. E. (2006). Co-operative inhibition by fluoride and zinc of glucosyl transferase production and polysaccharide synthesis by mutans streptococci in suspension cultures and biofilms. FEMS Microbiol. Lett. 254, 134–140, . doi: 10.1111/j.1574-6968.2005.00018.x. 16451191

[ref32] Koo H. Xiao J. Klein M. I. Jeon J. G. (2010). Exopolysaccharides produced by *Streptococcus mutans* glucosyltransferases modulate the establishment of microcolonies within multispecies biofilms. J. Bacteriol. 192, 3024–3032. doi: 10.1128/JB.01649-09, 20233920 PMC2901689

[ref33] Lemos J. A. Palmer S. R. Zeng L. Wen Z. T. Kajfasz J. K. Freires I. A. . (2019). The biology of *Streptococcus mutans*. Microbiol. Spectr. 7:GPP3-0051-2018. doi: 10.1128/microbiolspec.GPP3-0051-2018 PMC6615571 30657107

[ref34] Liu J. Ling J. Q. Zhang K. Huo L. J. Ning Y. (2012). Effect of sodium fluoride, ampicillin, and chlorhexidine on *Streptococcus mutans* biofilm detachment. Antimicrob. Agents Chemother. 56, 4532–4535. doi: 10.1128/AAC.00885-12, 22664966 PMC3421620

[ref35] Lorquin F. Piccerelle P. Orneto C. Robin M. Lorquin J. (2022). New insights and advances on pyomelanin production: from microbial synthesis to applications. J. Ind. Microbiol. Biotechnol. 49: kuac013. doi: 10.1093/jimb/kuac013, 35482661 PMC9338888

[ref36] Marquis R. E. (1995). Antimicrobial actions of fluoride for oral bacteria. Can. J. Microbiol. 41, 955–964. doi: 10.1139/m95-133, 7497353

[ref37] McLaughlin K. J. Strain-Damerell C. M. Xie K. Brekasis D. Soares A. S. Paget M. S. . (2010). Structural basis for NADH/NAD+ redox sensing by a rex family repressor. Mol. Cell 38, 563–575. doi: 10.1016/j.molcel.2010.05.006, 20513431

[ref38] Melkam A. Sionov R. V. Shalish M. Steinberg D. (2024). Enhanced anti-bacterial activity of arachidonic acid against the cariogenic bacterium *Streptococcus mutans* in combination with triclosan and fluoride. Antibiotics (Basel). 13:540. doi: 10.3390/antibiotics13060540 38927206 PMC11200779

[ref39] Mooney S. Leuendorf J. E. Hendrickson C. Hellmann H. (2009). Vitamin B6: a long known compound of surprising complexity. Molecules 14, 329–351. doi: 10.3390/molecules14010329, 19145213 PMC6253932

[ref40] Pagels M. Fuchs S. Pane-Farre J. Kohler C. Menschner L. Hecker M. . (2010). Redox sensing by a rex-family repressor is involved in the regulation of anaerobic gene expression in *Staphylococcus aureus*. Mol. Microbiol. 76, 1142–1161. doi: 10.1111/j.1365-2958.2010.07105.x, 20374494 PMC2883068

[ref41] Pitts N. B. Zero D. T. Marsh P. D. Ekstrand K. Weintraub J. A. Ramos-Gomez F. . (2017). Dental caries. Nat. Rev. Dis. Primers 3:17030. doi: 10.1038/nrdp.2017.30 28540937

[ref42] Rice K. C. Turner M. E. Carney O. V. Gu T. Ahn S. J. (2017). Modification of the *Streptococcus mutans* transcriptome by LrgAB and environmental stressors. Microb. Genom. 3:e000104. doi: 10.1099/mgen.0.000104, 28348880 PMC5361627

[ref43] Schneider-Rayman M. Steinberg D. Sionov R. V. Friedman M. Shalish M. (2021). Effect of epigallocatechin gallate on dental biofilm of *Streptococcus mutans*: an in vitro study. BMC Oral Health 21:447. doi: 10.1186/s12903-021-01798-4, 34525984 PMC8444437

[ref44] Seregina T. A. Shakulov R. S. Petrushanko I. Y. Lobanov K. V. Mironov A. S. (2025). Biosynthesis of ribose-5-phosphate-metabolic regulator of *Escherichia coli* viability. Cells 14:1775. doi: 10.3390/cells14221775, 41294827 PMC12651746

[ref45] Shen Y. Yu F. Qiu L. Gao M. Xu P. Zhang L. . (2022). Ecological influence by colonization of fluoride-resistant *Streptococcus mutans* in oral biofilm. Front. Cell. Infect. Microbiol. 12:1106392. doi: 10.3389/fcimb.2022.1106392 36699726 PMC9868560

[ref46] Shi D. Allewell N. M. Tuchman M. (2015). The N-Acetylglutamate synthase family: structures, function and mechanisms. Int. J. Mol. Sci. 16, 13004–13022. doi: 10.3390/ijms160613004, 26068232 PMC4490483

[ref47] Tinder E. L. Faustoferri R. C. Buckley A. A. Quivey R. G. Jr. Baker J. L. (2022). Analysis of the *Streptococcus mutans* proteome during acid and oxidative stress reveals modules of protein coexpression and an expanded role for the TreR transcriptional regulator. mSystems 7:e0127221. doi: 10.1128/msystems.01272-21, 35289653 PMC9040809

[ref48] Van Loveren C. (2001). Antimicrobial activity of fluoride and its in vivo importance: identification of research questions. Caries Res. 35, 65–70. doi: 10.1159/000049114 11359062

[ref49] Wang E. Bauer M. C. Rogstam A. Linse S. Logan D. T. von Wachenfeldt C. (2008). Structure and functional properties of the *Bacillus subtilis* transcriptional repressor rex. Mol. Microbiol. 69, 466–478. doi: 10.1111/j.1365-2958.2008.06295.x, 18485070

[ref50] Wu R. Tao Y. Cao Y. Zhou Y. Lin H. (2020). *Streptococcus mutans* membrane vesicles harboring glucosyltransferases augment *Candida albicans* biofilm development. Front. Microbiol. 11:581184. doi: 10.3389/fmicb.2020.581184 33042098 PMC7517897

[ref51] Xu X. Zhou X. D. Wu C. D. (2011). The tea catechin epigallocatechin gallate suppresses cariogenic virulence factors of *Streptococcus mutans*. Antimicrob. Agents Chemother. 55, 1229–1236. doi: 10.1128/AAC.01016-10, 21149622 PMC3067078

[ref52] Yu S. Ma Q. Li Y. Zou J. (2023). Molecular and regulatory mechanisms of oxidative stress adaptation in *Streptococcus mutans*. Mol Oral Microbiol 38, 1–8. doi: 10.1111/omi.12388, 36088636

[ref53] Zhang C. Kuang X. Zhou Y. Peng X. Guo Q. Yang T. . (2019). A novel small molecule, ZY354, inhibits dental caries-associated oral biofilms. Antimicrob. Agents Chemother. 63:e02414–18. doi: 10.1128/AAC.02414-18, 30858201 PMC6496107

[ref54] Zhang J. Lacroix C. Wortmann E. Ruscheweyh H. J. Sunagawa S. Sturla S. J. . (2019). Gut microbial beta-glucuronidase and glycerol/diol dehydratase activity contribute to dietary heterocyclic amine biotransformation. BMC Microbiol. 19:99. doi: 10.1186/s12866-019-1483-x, 31096909 PMC6524314

[ref55] Zheng X. Cheng X. Wang L. Qiu W. Wang S. Zhou Y. . (2015). Combinatorial effects of arginine and fluoride on oral bacteria. J. Dent. Res. 94, 344–353. doi: 10.1177/0022034514561259, 25477312 PMC4438734

