# Supplementary material for: Synergistic inhibition of Streptococcus mutans biofilms by fluoride and epigallocatechin gallate: insights from multi-omics analysis
Source: Front Microbiol. 2026 Feb 19;17:1766833. doi: 10.3389/fmicb.2026.1766833 (PMC12960562; doi:10.3389/fmicb.2026.1766833)
Supplement: Supplementary file 1 [file Data_Sheet_1.docx]

Supplementary Material

# Supplementary Data

**Material and Methods**

**Transcriptome analysis**

Total RNA was extracted and subjected to ribosomal RNA depletion, fragmentation, and cDNA synthesis using random hexamer primers with dUTP strand-specific labeling. Sequencing libraries were prepared via end repair, A-tailing, adapter ligation, and size selection, followed by quality assessment (Qubit, qPCR, Bioanalyzer). Sequencing was performed on the Illumina platform using sequencing-by-synthesis chemistry.

Raw reads were trimmed and filtered using fastp to remove adapter sequences and low-quality reads (Q20/Q30). Then clean reads were aligned to the S. mutans UA159 reference genome using Bowtie2 (v2.5.4). Rockhopper was used to identify novel transcripts, transcription start and termination sites (TSS/TTS), antisense RNAs, and operons. Promoter regions were predicted using a time-delay neural network (TDNN), and Shine-Dalgarno sequences and terminators were identified with RBSfinder and TransTermHP, respectively. Functional annotation and prediction of small non-coding RNAs were performed using RNAfold and IntaRNA.

Gene expression levels were normalized as FPKM and analyzed for differential expression using DESeq2 (for replicates; adjusted *p*, padj ≤ 0.05, |log2FC| ≥ 0) or edgeR (no replicates; padj ≤ 0.005, |log2FC| ≥ 1). Functional enrichment was performed using clusterProfiler to identify significant Gene Ontology (GO) terms and KEGG pathways.

**TMT labeling proteomic analyses**

Total protein was extracted from biofilm samples using SDT lysis buffer supplemented with DTT (1:100, v/v), followed by 5 min sonication on ice. Then, after centrifugation (12,000 × g, 15 min, 4°C), the supernatant was collected and incubated with sufficient IAM for 2 h. The mixture was centrifuged again (12,000 × g, 15 min, 4°C), and the pellet was washed with cold acetone and re-dissolved in DB buffer (8 M urea, 100 mM TEAB, pH 8.5). Protein concentrations were quantified using the BCA assay. Samples were digested overnight at 37°C in DB buffer with trypsin and TEAB. Lyophilized peptides were reconstituted in 0.1% formic acid and centrifuged (14,000 × g, 20 min, 4°C). A 200 ng aliquot of each clarified supernatant was loaded onto a Vanquish Neo UHPLC system (Thermo Fisher Scientific). Peptide separation was achieved using a reversed-phase C18 trap column (5 mm × 300 μm, 5 μm) and an analytical column (150 mm × 150 μm, 2 μm) at 50°C. Elution employed a linear gradient with solvent A (0.1% formic acid in water) and solvent B (80% acetonitrile with 0.1% formic acid).

Mass spectrometry was performed on an Orbitrap Astral mass spectrometer (Thermo Fisher Scientific) using data-independent acquisition (DIA) mode. MS1 spectra (m/z 380–980) were acquired at 240,000 resolution, and MS2 spectra (m/z 150–2000) at 80,000 resolution. Fragmentation utilized 300 variable isolation windows (2 Th) with an NCE of 25%. DIA data were processed using DIA-NN software with default parameters. Mass tolerances were 10 ppm (precursor) and 0.02 Da (fragment). Carbamidomethylation of cysteine was set as a fixed modification, and methionine oxidation and N-terminal acetylation were variable. Up to two missed cleavages were allowed. Peptides and proteins were filtered at a 1% FDR.

Protein quantification was conducted with thresholds of p < 0.05 and |log₂FC| above a defined cutoff. GO terms, KEGG pathway (*p* < 0.05) and differentially expressed proteins (DEPs, |fold change| > 1.2 or ＜0.83, *p* < 0.05) were visualized.

**Untargeted metabolomics analysis**

Biofilm samples were harvested, lyophilized, and extracted with ice-cold 80% methanol. Following centrifugation (15,000 × g, 15 min, 4°C), supernatants were diluted to 53% methanol and centrifuged again. Metabolites were separated using a Vanquish UHPLC system (Hypersil GOLD column, 100 × 2.1 mm, 1.9 μm) under a 12-min gradient at 0.2 mL/min with solvent A (0.1% formic acid in water) and solvent B (methanol). Mass spectrometry was performed on a Q Exactive HF/X instrument in polarity switching mode (±3.5 kV) with the following settings: capillary temperature 320°C, sheath gas 35 psi, and auxiliary gas 10 L/min.

Raw data were processed using XCMS for peak detection, alignment, and quantification. Metabolites were identified by spectral matching within a 10 ppm tolerance against KEGG, HMDB, and LIPIDMAPS databases. Quality control–based normalization (CV <30%) was applied prior to statistical analysis. Multivariate analyses included principal component analysis (PCA) and partial least squares discriminant analysis (PLS-DA) via the metaX platform, alongside univariate t-tests (VIP >1, *p* < 0.05, fold change ≥2 or ≤0.5). Differential metabolites were visualized using volcano plots and z-score normalized heatmaps. Pearson correlation and pathway enrichment analysis (x/n > y/N, p < 0.05) were performed to explore significant metabolic pathways and network alterations.

# Supplementary Figures and Tables

## Supplementary Figures


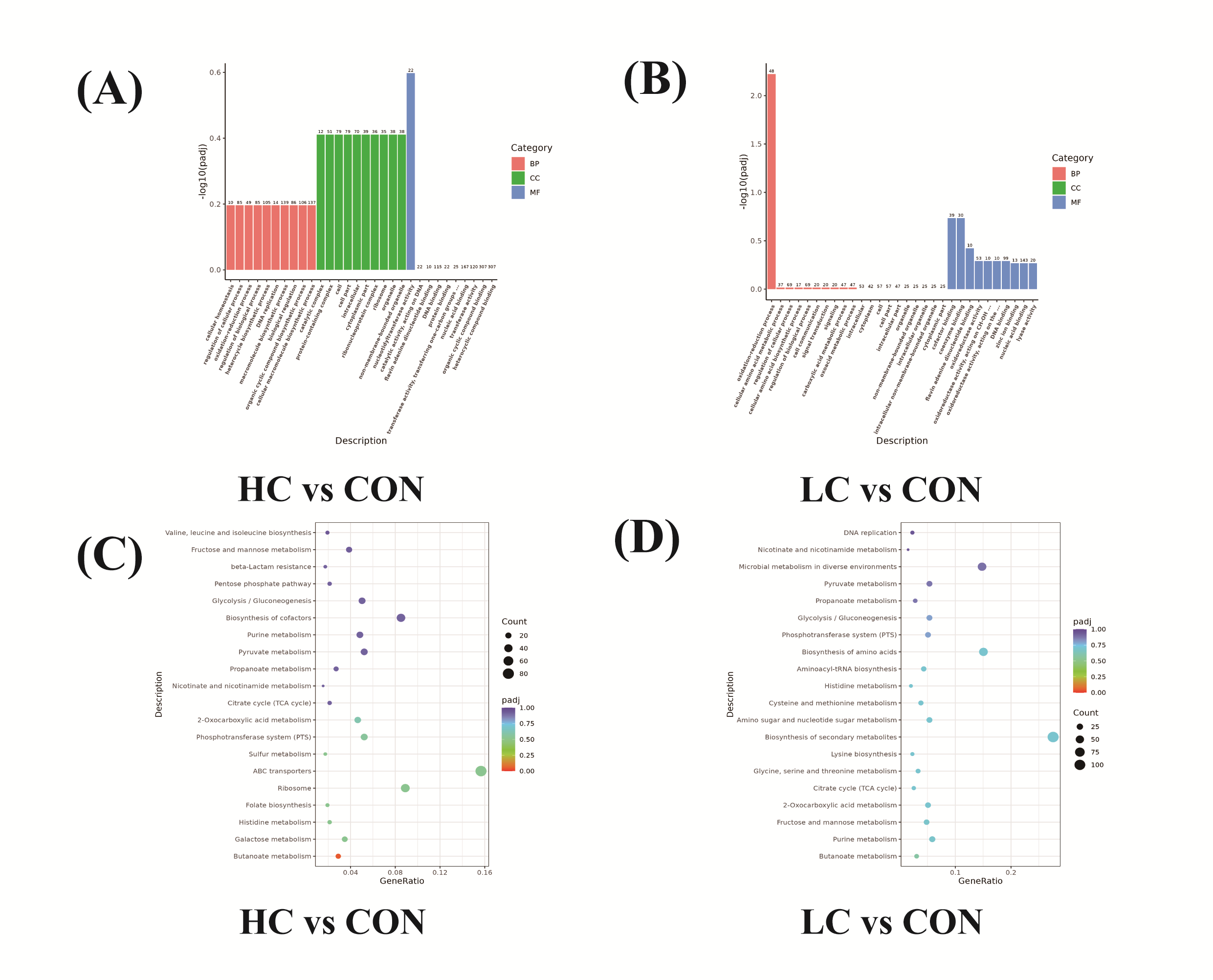


**Supplementary Figure 1: (A-B)** Transcriptome analysis. Top 30 enriched GO terms were displayed of different groups, and padj＜0.05 was regarded as significant different GO terms. **(C-D)** Top 20 KEGG pathways were showed, and padj＜0.05 was regarded as significant different KEGG pathways.


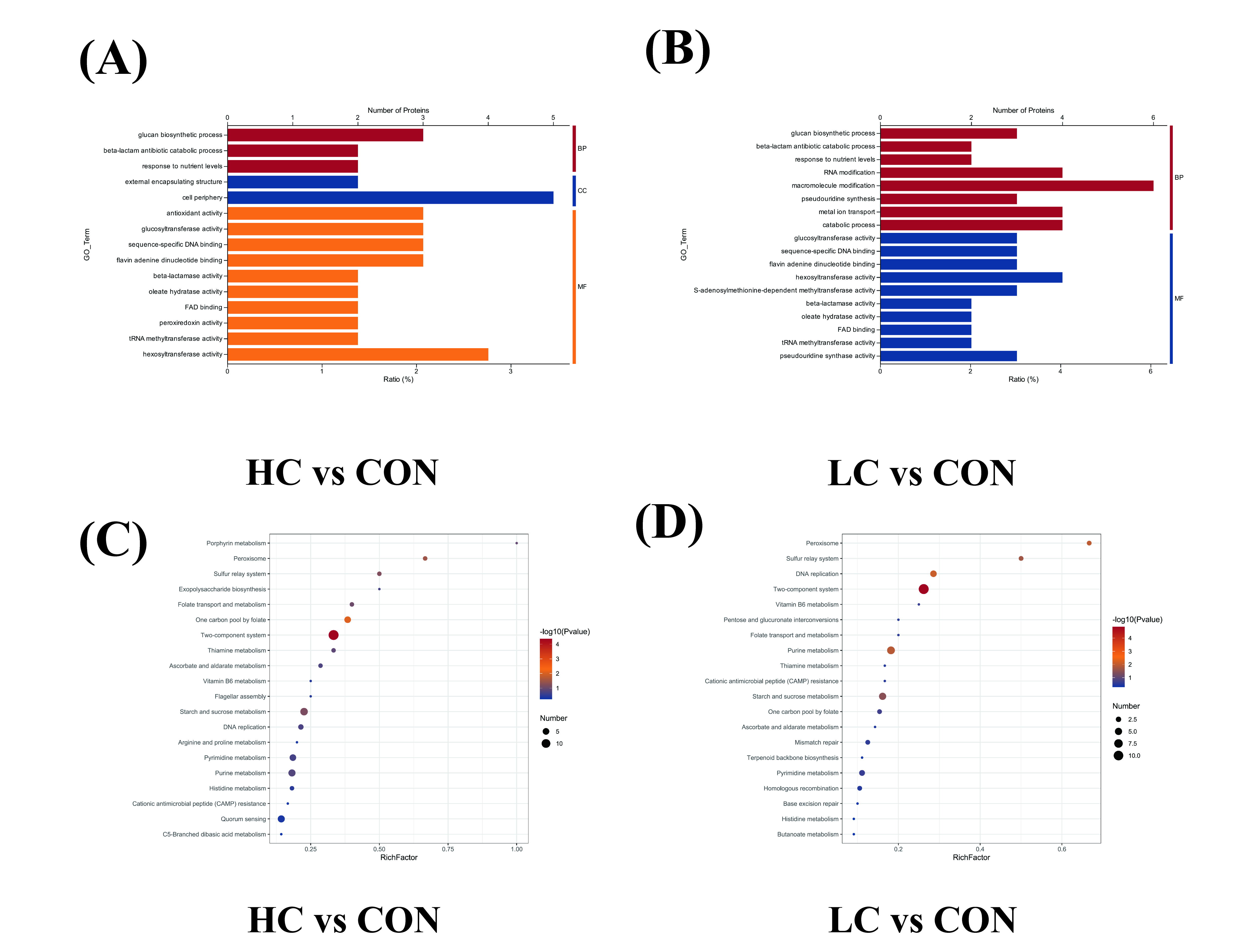


**Supplementary Figure 2:** **(A-B)** TMT labeling proteomic analysis. a-b Enriched GO terms were displayed of different groups, and *p*＜0.05 was regarded as significant different GO terms. **(C-D)** Top 20 KEGG pathways were showed, and *p*＜0.05 was regarded as significant different KEGG pathways.

## Supplementary Tables

Table S1 Significant GO term in HC vs. control comparison for TMT labeling proteomic analysis.

| GO ID | GO Term | GO Class | P value | Adjusted Pv | x | y | n | N | GO level |
| --- | --- | --- | --- | --- | --- | --- | --- | --- | --- |
| GO:0016209 | antioxidant activity | MF | 0.0038 | 0.3425 | 3 | 3 | 145 | 920 | 2 |
| GO:0009250 | glucan biosynthetic process | BP | 0.0038 | 0.3425 | 3 | 3 | 145 | 920 | 7 |
| GO:0046527 | glucosyltransferase activity | MF | 0.0038 | 0.3425 | 3 | 3 | 145 | 920 | 6 |
| GO:0043565 | sequence-specific DNA binding | MF | 0.0136 | 0.3425 | 3 | 4 | 145 | 920 | 5 |
| GO:0050660 | flavin adenine dinucleotide binding | MF | 0.0136 | 0.3425 | 3 | 4 | 145 | 920 | 5 |
| GO:0008800 | beta-lactamase activity | MF | 0.0247 | 0.3425 | 2 | 2 | 145 | 920 | 6 |
| GO:0030655 | beta-lactam antibiotic catabolic process | BP | 0.0247 | 0.3425 | 2 | 2 | 145 | 920 | 6 |
| GO:0050151 | oleate hydratase activity | MF | 0.0247 | 0.3425 | 2 | 2 | 145 | 920 | 6 |
| GO:0071949 | FAD binding | MF | 0.0247 | 0.3425 | 2 | 2 | 145 | 920 | 6 |
| GO:0051920 | peroxiredoxin activity | MF | 0.0247 | 0.3425 | 2 | 2 | 145 | 920 | 4 |
| GO:0008175 | tRNA methyltransferase activity | MF | 0.0247 | 0.3425 | 2 | 2 | 145 | 920 | 6 |
| GO:0030312 | external encapsulating structure | CC | 0.0247 | 0.3425 | 2 | 2 | 145 | 920 | 3 |
| GO:0031667 | response to nutrient levels | BP | 0.0247 | 0.3425 | 2 | 2 | 145 | 920 | 3 |
| GO:0016758 | hexosyltransferase activity | MF | 0.0248 | 0.3425 | 4 | 8 | 145 | 920 | 5 |
| GO:0071944 | cell periphery | CC | 0.0283 | 0.3738 | 5 | 12 | 145 | 920 | 3 |
| GO:0008757 | S-adenosylmethionine-dependent methyltransferase activity | MF | 0.0301 | 0.3738 | 3 | 5 | 145 | 920 | 6 |
| GO:0016741 | transferase activity, transferring one-carbon groups | MF | 0.0342 | 0.3738 | 9 | 29 | 145 | 920 | 4 |
| GO:0022857 | transmembrane transporter activity | MF | 0.0427 | 0.3738 | 20 | 83 | 145 | 920 | 3 |

x: Number of differentially expressed proteins (DEPs) associated with the specific GO term. y: Total number of background proteins associated with the same GO term. n: Number of DEPs annotated in the GO database. N: Total number of background proteins annotated in the GO database. GOlevl: Hierarchical level of the term within the GO database structure

Table S2 Significant GO term in LC vs. control comparison for TMT labeling proteomic analysis.

| GO ID | GO Term | GO Class | P value | Adjusted Pv | x | y | n | N | GO level |
| --- | --- | --- | --- | --- | --- | --- | --- | --- | --- |
| GO:0009250 | glucan biosynthetic process | BP | 0.0012 | 0.0829 | 3 | 3 | 99 | 920 | 7 |
| GO:0046527 | glucosyltransferase activity | MF | 0.0012 | 0.0829 | 3 | 3 | 99 | 920 | 6 |
| GO:0043565 | sequence-specific DNA binding | MF | 0.0045 | 0.1076 | 3 | 4 | 99 | 920 | 5 |
| GO:0050660 | flavin adenine dinucleotide binding | MF | 0.0045 | 0.1076 | 3 | 4 | 99 | 920 | 5 |
| GO:0016758 | hexosyltransferase activity | MF | 0.0063 | 0.1330 | 4 | 8 | 99 | 920 | 5 |
| GO:0008757 | S-adenosylmethionine-dependent methyltransferase activity | MF | 0.0103 | 0.1330 | 3 | 5 | 99 | 920 | 6 |
| GO:0008800 | beta-lactamase activity | MF | 0.0115 | 0.1330 | 2 | 2 | 99 | 920 | 6 |
| GO:0030655 | beta-lactam antibiotic catabolic process | BP | 0.0115 | 0.1330 | 2 | 2 | 99 | 920 | 6 |
| GO:0050151 | oleate hydratase activity | MF | 0.0115 | 0.1330 | 2 | 2 | 99 | 920 | 6 |
| GO:0071949 | FAD binding | MF | 0.0115 | 0.1330 | 2 | 2 | 99 | 920 | 6 |
| GO:0008175 | tRNA methyltransferase activity | MF | 0.0115 | 0.1330 | 2 | 2 | 99 | 920 | 6 |
| GO:0031667 | response to nutrient levels | BP | 0.0115 | 0.1330 | 2 | 2 | 99 | 920 | 3 |
| GO:0009451 | RNA modification | BP | 0.0159 | 0.1779 | 4 | 10 | 99 | 920 | 6 |
| GO:0043412 | macromolecule modification | BP | 0.0189 | 0.1802 | 6 | 21 | 99 | 920 | 5 |
| GO:0001522 | pseudouridine synthesis | BP | 0.0190 | 0.1802 | 3 | 6 | 99 | 920 | 7 |
| GO:0009982 | pseudouridine synthase activity | MF | 0.0190 | 0.1802 | 3 | 6 | 99 | 920 | 5 |
| GO:0030001 | metal ion transport | BP | 0.0317 | 0.2707 | 4 | 12 | 99 | 920 | 7 |
| GO:0016209 | antioxidant activity | MF | 0.0320 | 0.2707 | 2 | 3 | 99 | 920 | 2 |
| GO:0016780 | phosphotransferase activity, for other substituted phosphate groups | MF | 0.0320 | 0.2707 | 2 | 3 | 99 | 920 | 5 |
| GO:0009056 | catabolic process | BP | 0.0420 | 0.3374 | 4 | 13 | 99 | 920 | 4 |

Table S3 Significant KEGG term in HC vs. control comparison for TMT labeling proteomic analysis.

| MapID | MapTitle | P value | Adjusted Pv | x | y | n | N |
| --- | --- | --- | --- | --- | --- | --- | --- |
| map02020 | Two-component system | 0.0000 | 0.0021 | 14 | 42 | 74 | 681 |
| map00670 | One carbon pool by folate | 0.0085 | 0.2082 | 5 | 13 | 74 | 681 |
| map04146 | Peroxisome | 0.0325 | 0.5312 | 2 | 3 | 74 | 681 |

Table S4 Significant KEGG term in LC vs. control comparison for TMT labeling proteomic analysis.

| MapID | MapTitle | P value | Adjusted Pv | x | y | n | N |
| --- | --- | --- | --- | --- | --- | --- | --- |
| map02020 | Two-component system | 0.0000 | 0.0004 | 11 | 42 | 42 | 681 |
| map03030 | DNA replication | 0.0080 | 0.0967 | 4 | 14 | 42 | 681 |
| map04146 | Peroxisome | 0.0107 | 0.0967 | 2 | 3 | 42 | 681 |
| map00230 | Purine metabolism | 0.0121 | 0.0967 | 6 | 33 | 42 | 681 |
| map04122 | Sulfur relay system | 0.0206 | 0.1318 | 2 | 4 | 42 | 681 |
| map00500 | Starch and sucrose metabolism | 0.0359 | 0.1912 | 5 | 31 | 42 | 681 |

Table S5 Overlapped pathways and metabolites.

|  | Pathways | Metabolisms |
| --- | --- | --- |
| map00440 | Phosphonate and phosphinate metabolism | Foscarnet; Phosphoenolpyruvic acid |
| map00360 | Phenylalanine metabolism | 2-(2-Methylbenzamido)acetic acid; N-acetylphenylalanine; Capsaicin; D-(+)-Phenyllactic acid |
| map00030 | Pentose phosphate pathway | RIBOSE 5-PHOSPHATE; D-Ribose 1,5-bisphosphate |
| map00750 | Vitamin B6 metabolism | 3-Hydroxy-2-methylpyridine-4,5-dicarboxylate; Pyridoxal phosphate |
| map00592 | alpha-Linolenic acid metabolism | 9(S)-HpOTrE |
| map00790 | Folate biosynthesis | Neopterin; Dihydroneopterin phosphate |
| map04020 | Calcium signaling pathway | Cyclic ADP-ribose |
| map04918 | Thyroid hormone synthesis | RIBOSE 5-PHOSPHATE; NADPH |
| map00230 | Purine metabolism | RIBOSE 5-PHOSPHATE; Adenylosuccinic acid; inosine; Guanosine 5'-diphosphate |
| map00982 | Drug metabolism - cytochrome P450 | Aldophosphamide; Carboxyphosphamide |
| map00565 | Ether lipid metabolism | LysoPA(P-16:0/0:0) |
| map00590 | Arachidonic acid metabolism | (plusmn)5,6-DHET |
| map00730 | Thiamine metabolism | Pyridoxal phosphate |
| map03013 | RNA transport | Guanosine 5'-diphosphate |
| map04014 | Ras signaling pathway | Guanosine 5'-diphosphate |
| map04015 | Rap1 signaling pathway | Guanosine 5'-diphosphate |
| map04080 | Neuroactive ligand-receptor interaction | Thyrotropin-releasing hormone |
| map04144 | Endocytosis | Guanosine 5'-diphosphate |
| map04972 | Pancreatic secretion | Cyclic ADP-ribose |
| map00010 | Glycolysis / Gluconeogenesis | Phosphoenolpyruvic acid |
| map00250 | Alanine, aspartate and glutamate metabolism | N-Acetyl-L-aspartic acid; Adenylosuccinic acid |
| map04750 | Inflammatory mediator regulation of TRP channels | Capsaicin |
| map04921 | Oxytocin signaling pathway | Cyclic ADP-ribose |
| map04970 | Salivary secretion | Cyclic ADP-ribose |
| map04726 | Serotonergic synapse | (plusmn)5,6-DHET |

Table S6 Enriched pathways of multi-omics analysis in HC vs control comparison.

| Ion mode | Description | P value | Ratio | Count | Type | ID | KO |
| --- | --- | --- | --- | --- | --- | --- | --- |
| positive | Histidine metabolism | 0.0280 | 0.92 | 11 | Tran | SMU_RS05855/SMU_RS05830/SMU_RS05825/SMU_RS05845/SMU_RS05865/SMU_RS05860/SMU_RS00890/SMU_RS05835/SMU_RS05870/SMU_RS05820/SMU_RS05810 | smu00340 |
| positive | Histidine metabolism | 0.3398 | 0.18 | 2 | Prot | AAN58946.1, AAN58948.1 | map00340 |
| positive | Histidine metabolism | 1.0000 | 0.57 | 4 | Meta | trans-urocanate; L-Glutamic acid; 1-Methylhistidine; L-Histidine trimethylbetaine | map00340 |
| positive | Sulfur metabolism | 0.0621 | 0.90 | 9 | Tran | SMU_RS06655/SMU_RS03085/SMU_RS03090/SMU_RS07610/SMU_RS05970/SMU_RS00785/SMU_RS03095/SMU_RS02380/Novel00067 | smu00920 |
| positive | Sulfur metabolism | 0.6036 | 0.12 | 1 | Prot | AAN57933.1 | map00920 |
| positive | Sulfur metabolism | 1.0000 | 1.00 | 1 | Meta | SUCCINIC ACID | map00920 |
| positive | Cysteine and methionine metabolism | 0.8127 | 0.56 | 14 | Tran | SMU_RS05130/SMU_RS06655/SMU_RS04390/SMU_RS07605/SMU_RS07610/SMU_RS05400/SMU_RS07135/Novel00177/SMU_RS00340/SMU_RS00785/SMU_RS07175/SMU_RS04550/SMU_RS04440/SMU_RS02380 | smu00270 |
| positive | Cysteine and methionine metabolism | 0.7300 | 0.13 | 3 | Prot | AAN57933.1 AAN58656.1 AAN58864.1 | map00270 |
| positive | Cysteine and methionine metabolism | 0.4996 | 0.67 | 6 | Meta | S-adenosyl-L-methionine; Thiocysteine; 3-Sulfopyruvic acid; methionine sulfoxide; 5'-Methylthioadenosine; Glutathione | map00270 |
| positive | Phenylalanine, tyrosine and tryptophan biosynthesis | 0.9997 | 0.29 | 6 | Tran | SMU_RS03625/SMU_RS03630/SMU_RS03615/SMU_RS05870/SMU_RS03645/SMU_RS05940 | smu00400 |
| positive | Phenylalanine, tyrosine and tryptophan biosynthesis | 1.0000 | 0.11 | 2 | Prot | AAN58500.1, AAN59459.1 | map00400 |
| positive | Phenylalanine, tyrosine and tryptophan biosynthesis | 1.0000 | 0.60 | 3 | Meta | L-Arogenate; 4-Hydroxyphenylpyruvic acid; L-Phenylalanine | map00400 |
| negative | Histidine metabolism | 0.0280 | 0.92 | 11 | Tran | SMU_RS05855/SMU_RS05830/SMU_RS05825/SMU_RS05845/SMU_RS05865/SMU_RS05860/SMU_RS00890/SMU_RS05835/SMU_RS05870/SMU_RS05820/SMU_RS05810 | smu00340 |
| negative | Histidine metabolism | 0.3398 | 0.18 | 2 | Prot | AAN58946.1, AAN58948.1 | map00340 |
| negative | Histidine metabolism | 1.0000 | 0.50 | 3 | Meta | N-Carbamylglutamate; N-Formyl-L-aspartate; hydantoin-5-propionate | map00340 |
| negative | Sulfur metabolism | 0.0621 | 0.90 | 9 | Tran | SMU_RS06655/SMU_RS03085/SMU_RS03090/SMU_RS07610/SMU_RS05970/SMU_RS00785/SMU_RS03095/SMU_RS02380/Novel00067 | smu00920 |
| negative | Sulfur metabolism | 0.6036 | 0.13 | 1 | Prot | AAN57933.1 | map00920 |
| negative | Sulfur metabolism | 0.4821 | 1.00 | 1 | Meta | O-Succinyhomoserine | map00920 |
| negative | Starch and sucrose metabolism | 0.6720 | 0.60 | 26 | Tran | SMU_RS08435/SMU_RS04625/SMU_RS06980/SMU_RS06985/SMU_RS06990/SMU_RS04965/SMU_RS06975/SMU_RS09325/SMU_RS09335/SMU_RS01525/SMU_RS04525/SMU_RS09320/Novel00090/SMU_RS06970/SMU_RS04095/SMU_RS01585/SMU_RS07210/Novel00208/Novel00054/SMU_RS06995/SMU_RS05075/Novel00031/SMU_RS07245/Novel00159/SMU_RS07255/SMU_RS08440 | smu00500 |
| negative | Starch and sucrose metabolism | 0.0672 | 0.23 | 7 | Prot | AAN58619.1, AAN58705.1, AAN58706.1, AAN59189.1, AAN59238.1, AAN59631.1, AAN59642.1 | map00500 |
| negative | Starch and sucrose metabolism | 0.2310 | 1.00 | 2 | Meta | alpha-Maltose 1-phosphate; Trehalose | map00500 |
| negative | Purine metabolism | 0.2408 | 0.69 | 25 | Tran | SMU_RS04965/SMU_RS00225/SMU_RS00310/SMU_RS00365/SMU_RS09480/SMU_RS03160/SMU_RS00230/SMU_RS04820/SMU_RS03165/SMU_RS09345/SMU_RS04910/SMU_RS05670/SMU_RS00240/SMU_RS00320/SMU_RS00265/SMU_RS02295/Novel00010/SMU_RS06660/SMU_RS00255/SMU_RS00250/SMU_RS00325/SMU_RS00060/SMU_RS07805/SMU_RS05685/SMU_RS04835 | smu00230 |
| negative | Purine metabolism | 0.1576 | 0.18 | 6 | Prot | AAN57818.1, AAN57819.1, AAN57823.1, AAN57824.1, AAN57826.1, AAN59670.1 | map00230 |
| negative | Purine metabolism | 0.3283 | 0.64 | 9 | Meta | RIBOSE 5-PHOSPHATE; Deoxyinosine; Xanthosine; Phosphoribosyl formamidocarboxamide; dADP; Adenylosuccinic acid; 2'-Deoxyguanosine; inosine; Guanosine 5'-diphosphate | map00230 |
| negative | ABC transporters | 0.0613 | 0.69 | 81 | Tran | SMU_RS07565/SMU_RS05050/SMU_RS07570/SMU_RS02095/SMU_RS04890/SMU_RS01305/SMU_RS03085/SMU_RS07560/SMU_RS02100/SMU_RS05235/SMU_RS01300/SMU_RS01310/SMU_RS05220/SMU_RS07575/SMU_RS00910/SMU_RS03090/SMU_RS05225/SMU_RS05230/SMU_RS04630/SMU_RS04895/SMU_RS00905/SMU_RS04975/SMU_RS08825/SMU_RS05240/SMU_RS04100/SMU_RS04635/SMU_RS00900/SMU_RS04495/SMU_RS04970/SMU_RS03740/SMU_RS07120/SMU_RS03780/SMU_RS04310/SMU_RS03735/SMU_RS08935/SMU_RS04480/SMU_RS01295/SMU_RS09675/SMU_RS08830/SMU_RS08815/SMU_RS04315/SMU_RS09805/Novel00111/SMU_RS09680/SMU_RS04260/SMU_RS04305/SMU_RS04320/SMU_RS09800/SMU_RS03095/SMU_RS09810/SMU_RS04490/SMU_RS04255/SMU_RS06890/SMU_RS07115/Novel00091/SMU_RS07125/SMU_RS04080/SMU_RS05150/SMU_RS04485/SMU_RS04085/SMU_RS06895/SMU_RS01315/SMU_RS08605/SMU_RS06095/SMU_RS08655/SMU_RS04090/SMU_RS05995/Novel00067/SMU_RS09690/SMU_RS04200/SMU_RS08810/SMU_RS08375/SMU_RS06100/SMU_RS04195/Novel00080/SMU_RS02210/SMU_RS09685/SMU_RS09070/SMU_RS05155/Novel00107/SMU_RS03110 | smu02010 |
| negative | ABC transporters | 0.7353 | 0.12 | 12 | Prot | AAN57957.1, AAN57959.1, AAN58183.1, AAN58676.1, AAN58677.1, AAN58760.1, AAN58901.1, AAN59434.1, AAN59435.1, AAN59436.1, AAN59451.1, AAN59497.1 | map02010 |
| negative | ABC transporters | 0.6096 | 0.67 | 2 | Meta | D-Mannitol; Trehalose | map02010 |
| negative | Phenylalanine, tyrosine and tryptophan biosynthesis | 0.9997 | 0.29 | 6 | Tran | SMU_RS03625/SMU_RS03630/SMU_RS03615/SMU_RS05870/SMU_RS03645/SMU_RS05940 | smu00400 |
| negative | Phenylalanine, tyrosine and tryptophan biosynthesis | 1.0000 | 0.11 | 2 | Prot | AAN58500.1, AAN59459.1 | map00400 |
| negative | Phenylalanine, tyrosine and tryptophan biosynthesis | 0.0244 | 1.00 | 5 | Meta | D-Erythrose 4-phosphate; Phenylpyruvic acid; Fosfructose; Phosphoenolpyruvic acid; Shikimate | map00400 |

Table S7 Enriched pathways of multi-omics analysis in LC vs control comparison.

| Ion mode | Description | P  value | Ratio | Count | Type | ID | KO |
| --- | --- | --- | --- | --- | --- | --- | --- |
| positive | Purine metabolism | 0.0311 | 0.64 | 23 | Tran | SMU_RS03160/SMU_RS00325/SMU_RS03165/SMU_RS00365/SMU_RS04965/SMU_RS00320/SMU_RS00240/SMU_RS04820/SMU_RS07805/SMU_RS09345/SMU_RS00230/SMU_RS00255/SMU_RS00265/SMU_RS00250/SMU_RS06660/SMU_RS00225/Novel00183/Novel00006/SMU_RS09845/SMU_RS00060/SMU_RS05960/Novel00010/SMU_RS00310 | smu00230 |
| positive | Purine metabolism | 0.0121 | 0.18 | 6 | Prot | AAN57818.1 AAN57819.1 AAN57823.1 AAN57824.1 AAN57837.1 AAN59670.1 | map00230 |
| positive | Purine metabolism | 0.7308 | 0.40 | 4 | Meta | 5-Hydroxyisourate; 2'-Deoxyadenosine; Adenosine; Guanosine | map00230 |
| positive | Pyrimidine metabolism | 0.9615 | 0.33 | 10 | Tran | SMU_RS03160/SMU_RS03165/SMU_RS07380/SMU_RS03970/SMU_RS03290/SMU_RS01595/SMU_RS05740/SMU_RS05175/SMU_RS05630/SMU_RS08470 | smu00240 |
| positive | Pyrimidine metabolism | 0.2285 | 0.11 | 3 | Prot | AAN58649.1, AAN58909.1 AAN59670.1 | map00240 |
| positive | Pyrimidine metabolism | 0.6848 | 0.43 | 3 | Meta | 2'-Deoxycytidine; 2'-Deoxyuridine; pseudouridine | map00240 |
| negative | Purine metabolism | 0.0311 | 0.64 | 23 | Tran | SMU_RS03160/SMU_RS00325/SMU_RS03165/SMU_RS00365/SMU_RS04965/SMU_RS00320/SMU_RS00240/SMU_RS04820/SMU_RS07805/SMU_RS09345/SMU_RS00230/SMU_RS00255/SMU_RS00265/SMU_RS00250/SMU_RS06660/SMU_RS00225/Novel00183/Novel00006/SMU_RS09845/SMU_RS00060/SMU_RS05960/Novel00010/SMU_RS00310 | smu00230 |
| negative | Purine metabolism | 0.0121 | 0.18 | 6 | Prot | AAN57818.1, AAN57819.1, AAN57823.1, AAN57824.1, AAN57837.1, AAN59670.1 | map00230 |
| negative | Purine metabolism | 0.5610 | 0.43 | 6 | Meta | RIBOSE 5-PHOSPHATE; 5-Amino-1-(5-phospho-D-ribosyl)imidazole-4-carboxylic acid; Adenylosuccinic acid; inosine; Inosinic acid; Guanosine 5'-diphosphate | map00230 |
| negative | Pyrimidine metabolism | 0.9615 | 0.33 | 10 | Tran | SMU_RS03160/SMU_RS03165/SMU_RS07380/SMU_RS03970/SMU_RS03290/SMU_RS01595/SMU_RS05740/SMU_RS05175/SMU_RS05630/SMU_RS08470 | smu00240 |
| negative | Pyrimidine metabolism | 0.2285 | 0.11 | 3 | Prot | AAN58649.1, AAN58909.1, AAN59670.1 | map00240 |
| negative | Pyrimidine metabolism | 1.0000 | 0.40 | 2 | Meta | dCMP; Uridine | map00240 |
|  |  |  |  |  |  |  |  |
